# Supplementary material for: METTL3 drives NAFLD-related hepatocellular carcinoma and is a therapeutic target for boosting immunotherapy
Source: Cell Rep Med. 2023 Aug 15;4(8):101144. doi: 10.1016/j.xcrm.2023.101144 (PMC10439254; doi:10.1016/j.xcrm.2023.101144)
Supplement: Document S1. Figures S1–S7 and Table S1 [file mmc1.pdf]

Cell Reports Medicine, Volume *4*

Supplemental information

METTL3 drives NAFLD-related hepatocellular carcinoma and is a therapeutic target for boosting immunotherapy

Yasi Pan, Huarong Chen, Xiang Zhang, Weixin Liu, Yanqiang Ding, Dan Huang, Jianning Zhai, Wenchao Wei, Jun Wen, Danyu Chen, Yunfei Zhou, Cong Liang, Nathalie Wong, Kwan Man, Alvin Ho-Kwan Cheung, Chi Chun Wong, and Jun Yu

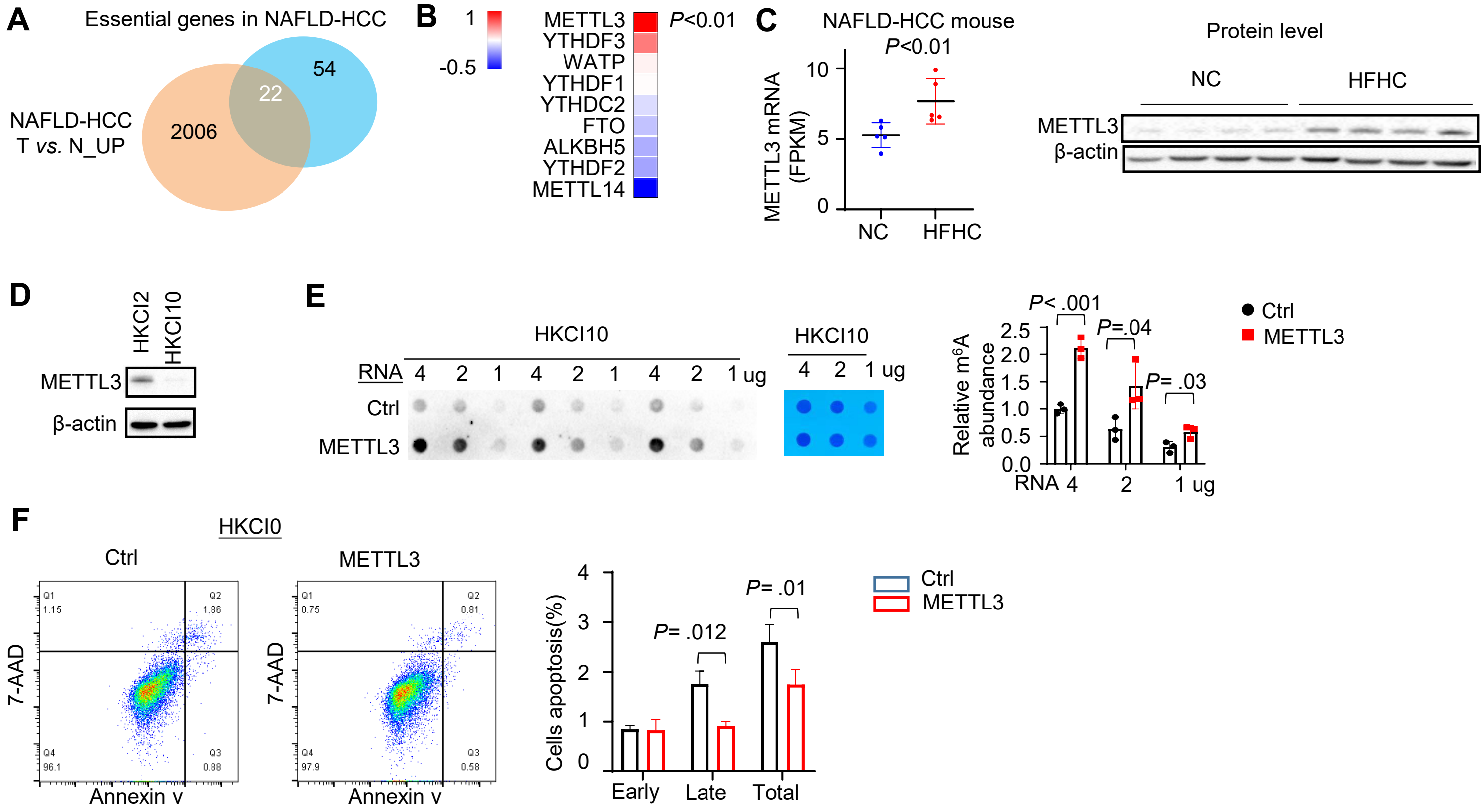

Figure S1. METTL3 is up-regulated in NAFLD-HCC patients and NAFLD-HCC mouse model. Related to Figure 1. (**A**) Integrated analysis of genes 1) increased in NAFLD-HCC in RNA-seq of 17 paired NAFLD-HCC and non-tumor tissues and 2) essential for NAFLD-HCC growth; (**B**) METTL3 is the top up-regulated m^{6}A regulator in 17 paired NAFLD-HCC patients; (**C**) METTL3 mRNA was up-regulated in dietary (HFHC) treated NAFLD-HCC animal model by RNA-seq, METTL3 protein was also upregulated. (**D**) Basal protein levels of METTL3 in two NAFLD-HCC cell lines. (**E**) Dot blot quantification of m^{6}A abundance in mRNA transcripts in HKCI10 cells overexpressing METTL3 (*left*). MB was used as a loading control (*middle*). Relative m^{6}A abundance in mRNA transcripts in HKCI10 cells (*right*). (**F**) METTL3 overexpression decreased apoptosis in HKCI10 cells as determined by annexin V–phycoerythrin and 7-AAD staining and flow cytometry. Data are presented as mean ± SD. Statistical significance was determined by one-way ANOVA or two-sided Student's *t*-test where appropriate.

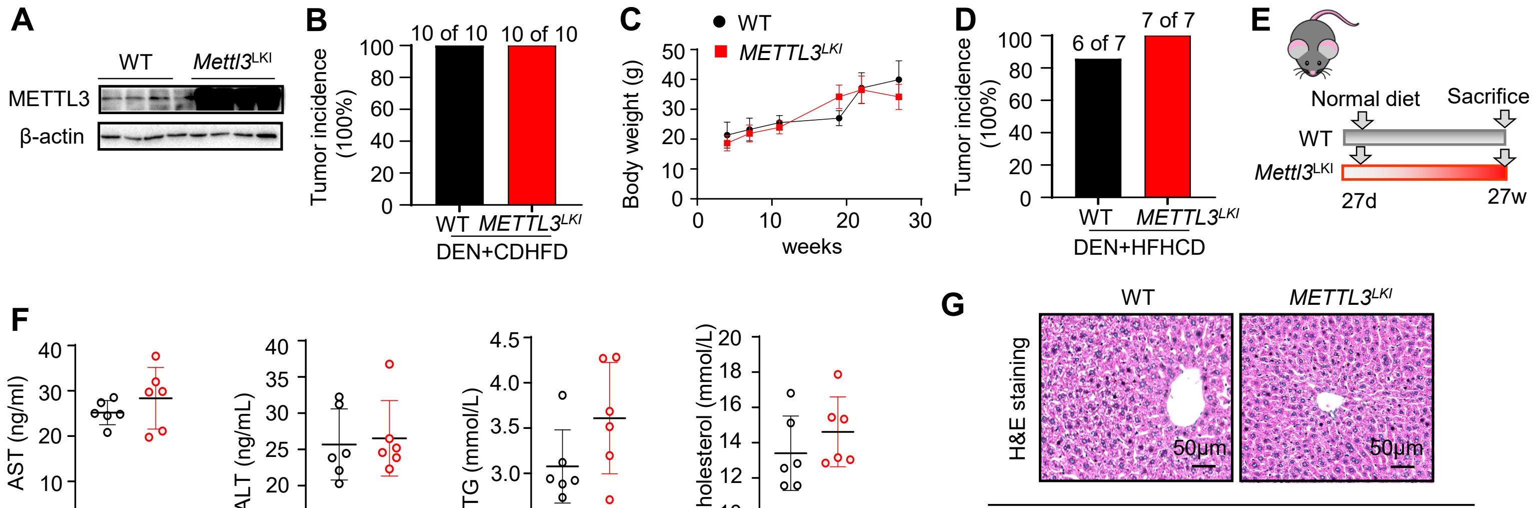

Histological scoring	WT	METTL3LKI
Steatosis	0	0
Inflammation	0.02±0.12	0.02±0.08

Figure S2. Hepatocyte-specific METTL3 knockin accelerates diet-induced NAFLD-HCC. Related to Figure 2. (**A**) Western blot confirmed overexpression of METTL3 in the livers of *Mettl3*^{LKI} mice. (**B**) NAFLD-HCC tumor incidence in WT and *METTL3*^{LKI} mice treated with DEN plus CDHFD. (**C**) Body weight development in WT and *METTL3*^{LKI} mice treated with DEN plus CDHFD. (**D**) NAFLD-HCC tumor incidence in WT and *METTL3*^{LKI} mice treated with DEN plus HFHC diet. (**E**) Experimental schematic of WT and *METTL3*^{LKI} mice were fed with normal diet for 27 weeks (n=6/group) . (**F**) Serum AST, ALT, TG and cholesterol level in WT and *METTL3*^{LKI} mice treated with normal diet at 27 weeks. (**G**) Representative images of H&E staining in WT and *METTL3*^{LKI} mice fed normal diet at 27 weeks. Histological scoring in WT and *METTL3*^{LKI} mice fed normal diet at 27 weeks. Data are presented as mean ± SD. Statistical significance was determined by two-sided Student's *t*-test.

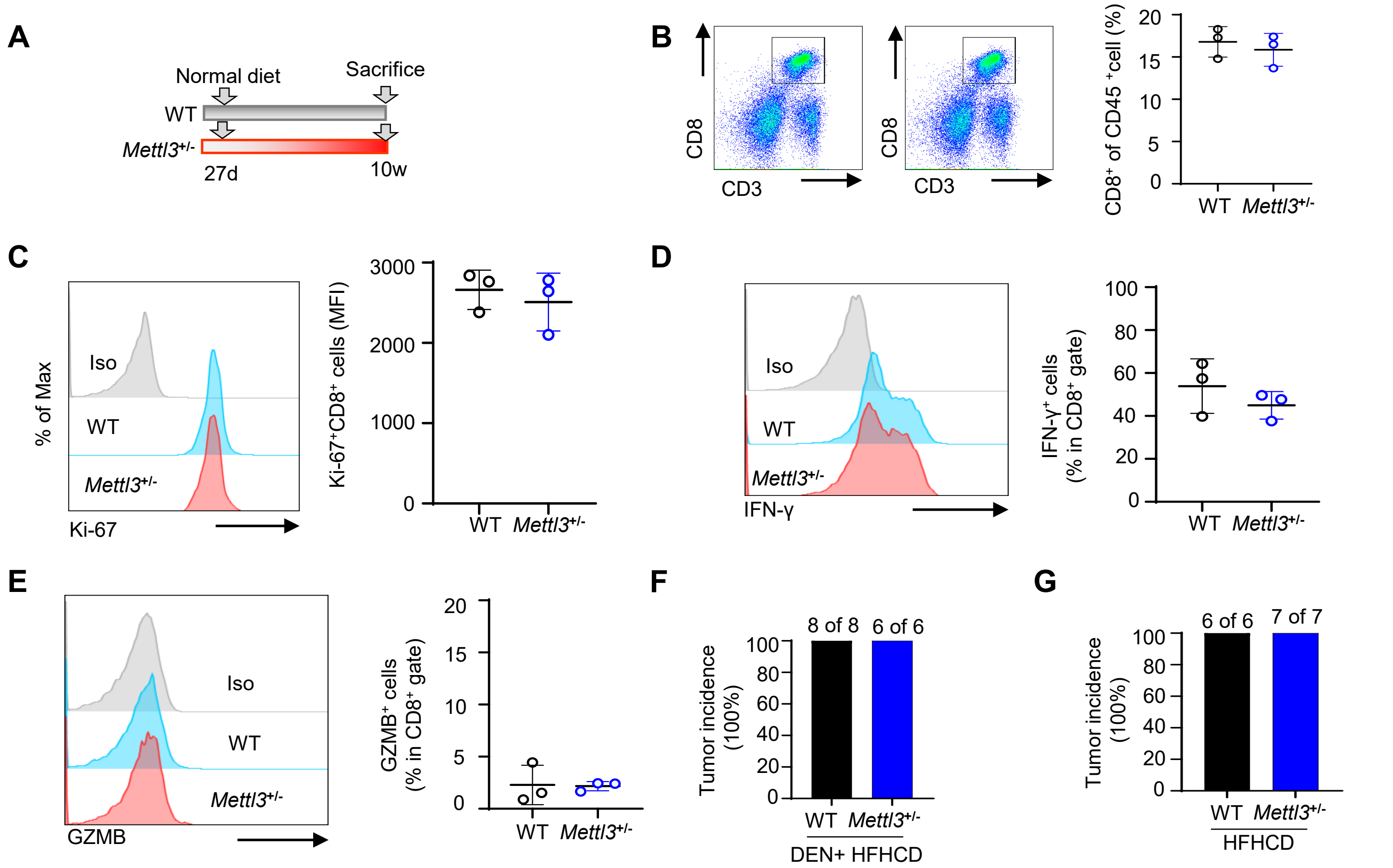

Figure S3. METTL3 knockout suppresses diet-induced NAFLD-HCC. Related to Figure 3. (**A**) Experimental schematic of WT and *Mettl3*$^{+/-}$ mice were fed with normal diet (ND) for 10 weeks (n=3/group). (**B**) Infiltration of CD8^{+} T cells in spleen from WT and *Mettl3*$^{+/-}$ mice treated with ND by flow cytometry. (**C**) Infiltration of ki-67^{+} CD8^{+} T cells in spleen from WT and *Mettl3*$^{+/-}$ mice treated with ND by flow cytometry. (**D and E**) Infiltration of IFN-γ^{+} (D) and GZMB^{+} (E) CD8^{+} T cells in spleen from WT and *Mettl3*$^{+/-}$ mice fed ND by flow cytometry.(**F**) NAFLD-HCC tumor incidence in WT and *Mettl3*$^{+/-}$ mice treated with DEN plus HFHCD. (**G**) NAFLD-HCC tumor incidence in WT and *Mettl3*$^{+/-}$ mice treated with HFHCD. Data are presented as mean ± SD. Statistical significance was determined by two-sided Student's *t*-test.

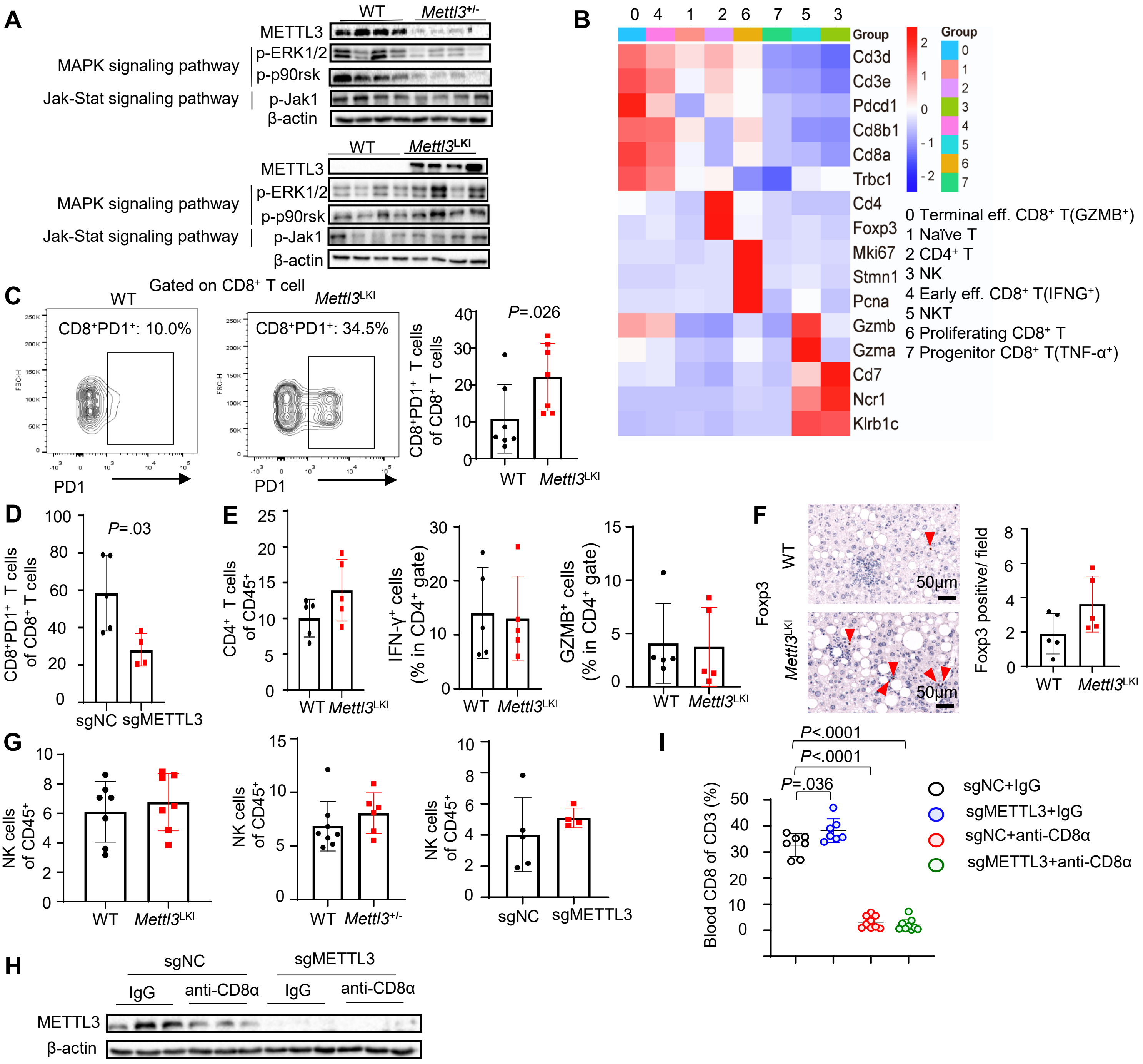

Figure S4. Tumor-intrinsic METTL3 restricts the activation and effector states of CD8⁺ T cells. Related to Figure 4. (**A**) Detection of MAPK signaling pathway and Jak-Stat signaling pathway by Western blot in WT and *Mettl3*$^{+/-}$ mice fed HFHCD (*upper*), also WT and *Mettl3*^{LKI} mice treated with DEN plus CDHFD (*bottom*). (**B**) Gene markers for different clusters of tumor-infiltrating T/NK-cells population. (**C**) Proportion of $CD8^+$ $PD1^+$ T cells (among $CD8^+$ T cells) in WT and *Mettl3*^{LKI} mice treated with DEN plus HFHCD by flow cytometry. (**D**) Proportion of $CD8^+$ $PD1^+$ T cells (among $CD8^+$ T cells) in NAFLD-HCC orthotopic mice model with sgNC and sgMETTL3 in Hepa1-6 by flow cytometry. (**E**) Proportion of $CD4^+$ T cells (among $CD45^+$ T cells) and its function markers (among $CD4^+$ T cells) in tumors from WT and *Mettl3*^{LKI} mice treated with DEN plus CDHFD by flow cytometry. (**F**) Foxp3 staining of livers of WT and *Mettl3*^{LKI} mice. (**G**)Tumor infiltration of NK cells in different models by flow cytometry, WT and *Mettl3*^{LKI} mice treated with DEN plus CDHFD (*left*), WT and *Mettl3*$^{+/-}$ mice treated with DEN plus HFHCD (*middle*), NAFLD-HCC orthotopic mice model with sgNC and sgMETTL3 in Hepa1-6 by flow cytometry (*right*). (**H**) Knockdown efficiency of METTL3 in orthotopic tumors by western blot. (**I**) Proportion of $CD8^+$ T cells (among $CD3^+$ T cells) in blood by flow cytometry. Successfully depletion of $CD8^+$ T cells using anti-CD8α compared to IgG group. Data are presented as mean ± SD. Statistical significance was determined by one-way ANOVA or two-sided Student's *t*-test where appropriate.

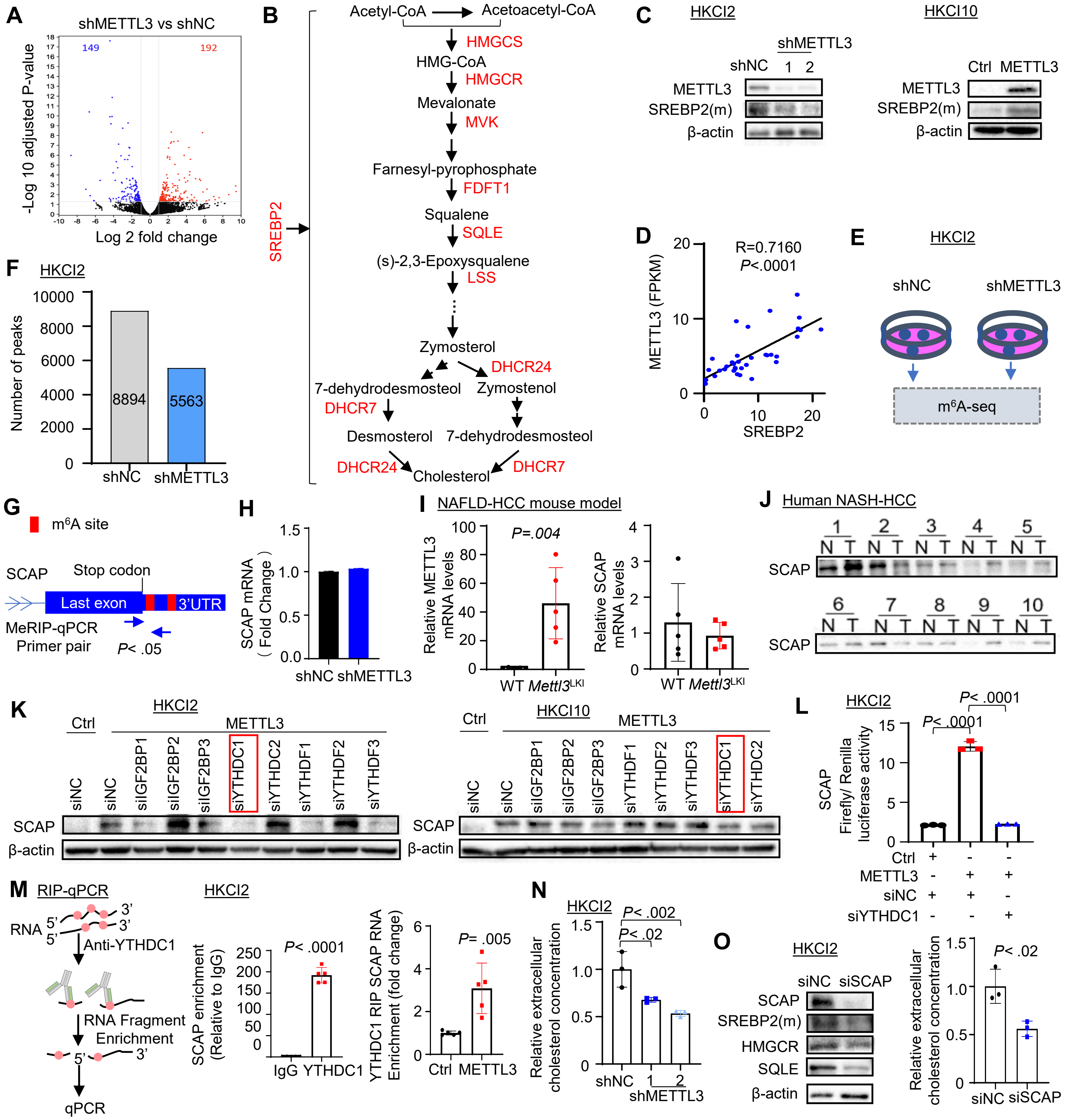

Figure S5. SCAP serves as a target of METTL3 via an YTHDC1-dependent mechanism in NAFLD-HCC. Related to Figure 5 and Table S1. (**A**) RNA-seq showed that 149 genes were down-regulated when METTL3 was silenced in HKCI2 cells. (**B**) Cholesterol biosynthesis pathway related genes were down regulated when METTL3 knockdown in HKCI2 cells by RNA-seq (in red). (**C**) Mature SREBP2 protein expression in HKCI2 cells with METTL3 knockdown, and HKCI10 cells with METTL3 overexpression. (**D**) Correlation of METTL3 with SREBP2 in human NAFLD-HCC. (**E**) Flow chart for m^{6}A-seq of NAFLD-HCC cells (HKCI2) with or without METTL3 knockdown. (**F**) m^{6}A-seq of HKCI2 showed a number of m^{6}A peaks. (**G**) Scheme showing the design of primers for MeRIP-qPCR to validate m^{6}A modifications on SCAP mRNA. Potential m^{6}A sites were highlighted in red, from 47413830 to 47413930 (*P*< .05 in m^{6}A-seq), and 94419499 to 47419600 (*P*>.05). Primers used in MeRIP-qPCR to validated m^{6}A modification are indicated by arrows in blue. (**H**) No change in SCAP mRNA by METTL3 knockdown in HKCI2 cells by RNA-seq. (**I**) METTL3 mRNA level was upregulated in *METTL3*^{LKI} mice compared to WT mice by qPCR, while SCAP mRNA expression was unchanged. (**J**) SCAP protein expression in human NAFLD-HCC was validated in an independent cohort (n=10). Loading control is identical to Figure 1C. (**K**) Screening potential m^{6}A reader for SCAP mediated by METTL3 in HKCI2 and HKCI10 by western blot. (**L**) Luciferase activity of SCAP 3-UTR in HKCI2 cells upon METTL3 overexpression with or without siYTHDC1, relative to Renilla luciferase activity. (**M**) YTHDC1-RIP-qPCR in HKCI2 cells. Schematic of YTHDC1-RIP-qPCR in HKCI2 cells (*left*). Relative enrichment of SCAP RNA by YTHDC1 in HKCI2 cells (*middle*). Relative enrichment of SCAP upon METTL3 overexpression in HKCI2 by YTHDC1-RIP-qPCR (*right*). (**N**) Relative extracellular cholesterol concentration upon METTL3 knockdown. (**O**) Cholesterol biosynthesis genes protein was suppressed in HKCI2-siSCAP cells (*left*), and relative extracellular cholesterol concentration upon SCAP knockdown (*right*) in HKCI2. Data are presented as mean ± SD. Statistical significance was determined by one-way ANOVA or two-sided Student's *t*-test where appropriate.

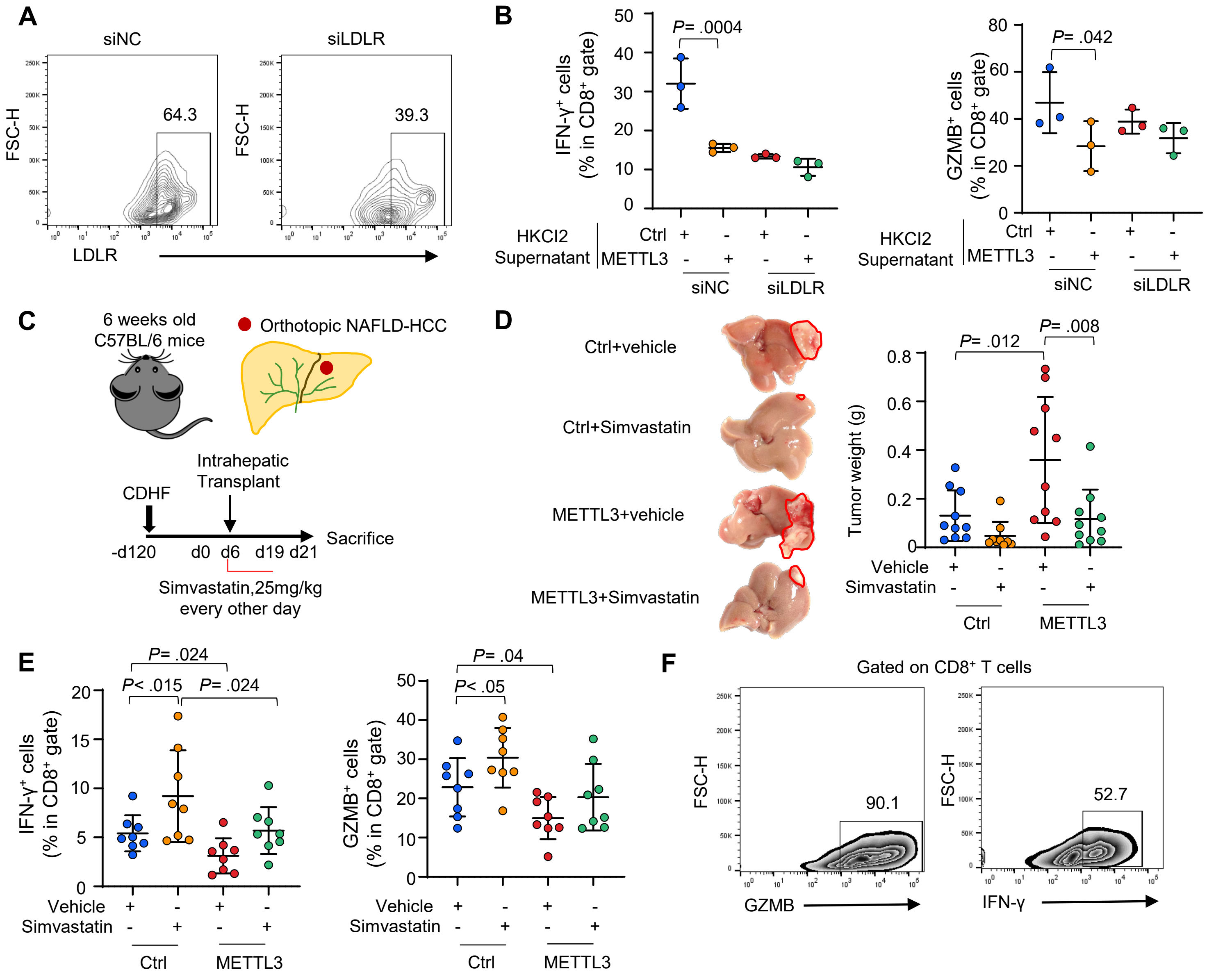

Figure S6. METTL3-induced cholesterol functions as an inducer of immunosuppression in NAFLD-HCC microenvironment. Related to Figure 6 and Table S1. (**A**) Knockdown efficiency of siLDLR in human T cells by flow cytometry. (**B**) Quantification of human IFN-γ$^+$ and GZMB$^+$ CD8$^+$ T cells from different groups by flow cytometry. (**C**) Experimental design for the orthotopic NAFLD-HCC model and simvastatin treatment. (**D**) Representative images of mouse livers and tumor weight at end point (n=10/group) . (**E**) Quantification of tumor-infiltrating IFN-γ$^+$ and GZMB$^+$ CD8$^+$ T cells. (**F**) Expression of IFN-γ$^+$ and GZMB$^+$ CD8$^+$ T cells by flow cytometry before coculture. Data are presented as mean ± SD. Statistical significance was determined by one-way ANOVA or two-sided Student's *t*-test where appropriate.

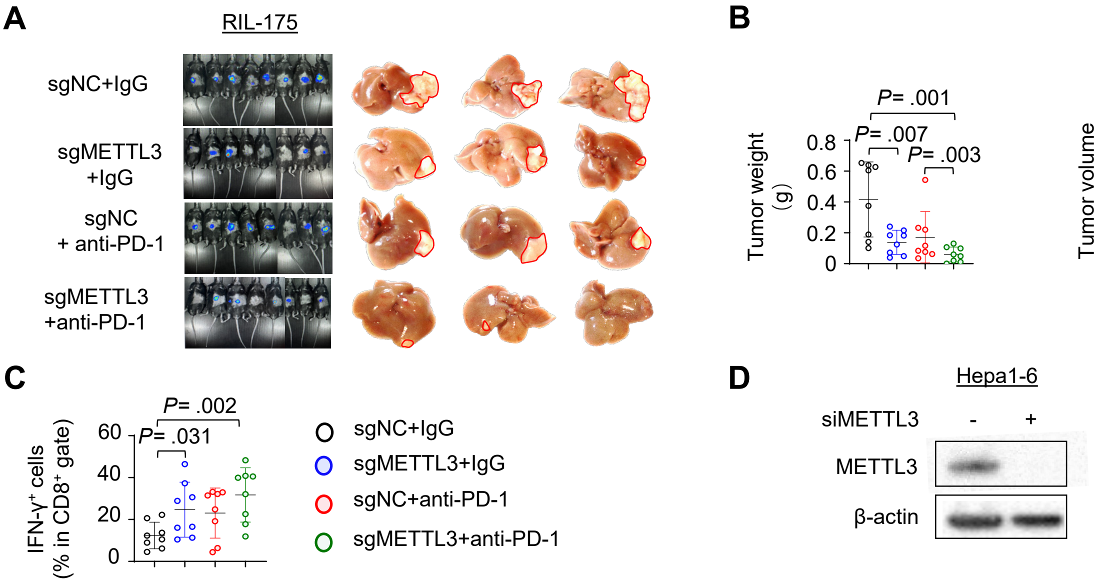

Figure S7. METTL3 is a therapeutic target for NAFLD-HCC. Related to Figure 7. (A) C57BL/6J mice were inoculated with 5x10^5 RIL175-sgNC and RIL175-sgMETTL3 cells, and then treated with anti-PD1 or IgG control. Bioluminescent imaging and the representative gross morphology of livers. **(B)** Tumor weight (left) and volume (right) at end point (n=8/group). **(C)** Quantification of tumor-infiltrating IFN-γ$^+$ CD8$^+$ T cell from different groups. **(D)** Knockdown efficiency of METTL3 in Hepa1-6 by nanoparticle siRNA was determined by western blot. Data are presented as mean ± SD. Statistical significance was determined by one-way ANOVA or two-sided Student's *t*-test where appropriate.

Table S1. Oligonucleotides used in this study. Related to STAR Methods.

REAGENT or RESOURCE	SOURCE	IDENTIFIER
Oligonucleotides		
Human SCAP for Merip-qPCR<br>Forward: TGGTGTATGTGCCCTCTGTG<br>Reverse: CCCCAAGTCCAGGTTCAGT	This paper	In this study
Human β-actin for RT-PCR<br>Forward: CACCATTGGCAATGAGCGGTTC<br>Reverse: AGGTCTTTGCGGATGTCCACGT	This paper	In this study
Mouse β-actin for RT-PCR<br>Forward: CATTGCTGACAGGATGCAGAAGG<br>Reverse: TGCTGGAAGGTGGACAGTGAGG	This paper	In this study
Human METTL3 for RT-PCR<br>Forward: CTATCTCCTGGCACTCGCAAGA<br>Reverse: GCTTGAACCGTGCAACCACATC	This paper	In this study
Mouse Mettl3 for RT-PCR<br>Forward: CAGTGCTACAGGATGACGGCTT<br>Reverse: CCGTCCTAATGATGCGCTGCAG	This paper	In this study
SiRNA for YTHDF1-1<br>Forward: CCUCCACCCAUAAAGCAUATT<br>Reverse: UAUGCUUUAUGGGUGGAGGTT	This paper	In this study
SiRNA for YTHDF1-2<br>Forward: GCUCCAUUAAGUACUCCAUTT<br>Reverse: AUGGAGUACUUAAUGGAGCTT	This paper	In this study
SiRNA for YTHDF2-1<br>Forward: GCUCUGGAUAUAGUAGCAATT<br>Reverse: UUGCUACUAUAUCCAGAGCTT	This paper	In this study
SiRNA for YTHDF2-2<br>Forward: GCGGGUCCAUUACUAGUAATT<br>Reverse: UUACUAGUAAUGGACCCGCTT	This paper	In this study
SiRNA for YTHDF3-1<br>Forward: GGGACAAUCAACACAAAGUTT<br>Reverse: ACUUUGUGUUGAUUGUCCCTT	This paper	In this study
SiRNA for YTHDF3-2<br>Forward: GACUAGCAUUGCAACCAAUTT<br>Reverse: AUUGGUUGCAAUGCUAGUCTT	This paper	In this study
SiRNA for YTHDC1-1<br>Forward: GCUCUGCAUCAGAGUCAUATT<br>Reverse: UAUGACUCUGAUGCAGAGCTT	This paper	In this study
SiRNA for YTHDC1-2<br>Forward: GCAAGGAGUGUUAUCUUAATT<br>Reverse: UUAAGAUAACACUCCUUGCTT	This paper	In this study
SiRNA for YTHDC2-1<br>Forward: GCAGAGAACUGUUCUAAAUTT<br>Reverse: AUUUAGAACAGUUCUCUGCTT	This paper	In this study
SiRNA for YTHDC2-2<br>Forward: CCUGUUCGAUACUUCAUAATT<br>Reverse: UUAUGAAGUAUCGAACAGGTT	This paper	In this study
SiRNA for IGF2BP1-1<br>Forward: CCCAGUAUGUGGGUGCCAUTT<br>Reverse: AUGGCACCCACAUACUGGGTT	This paper	In this study
SiRNA for IGF2BP1-2<br>Forward: CCAAAGUUCGUAUGGUUAUTT<br>Reverse: AUAACCAUACGAACUUUGGTT	This paper	In this study
SiRNA for IGF2BP2-1<br>Forward: GUCAACGUCACAUAUGCAATT<br>Reverse: UUGCAUAUGUGACGUUGACTT	This paper	In this study

SiRNA for IGF2BP2-2<br>Forward: GAGGGCUUGACCAUAAAGATT<br>Reverse: UCUUUAUGGUCAAGCCCUCTT	This paper	In this study
SiRNA for IGF2BP3-1<br>Forward: GGAUUCGGAAACUUCAGAUTT<br>Reverse: AUCUGAAGUUUCCGAAUCCTT	This paper	In this study
SiRNA for IGF2BP3-2<br>Forward: CACCUUGAAAGUAGCCUAUTT<br>Reverse: AUAGGCUACUUUCAAGGUGTT	This paper	In this study
Mouse Mettl3 siRNA<br>Forward: CCAAGGAAGAGUGCAUGAATT<br>Reverse: ACUCUUCCUUGGTT	This paper	In this study
SiNC:<br>Forward: UUCUCCGAACGUGUCACGUTT<br>Reverse: ACGUGACACGUUCGGAGAATT	This paper	In this study
Negative control siRNA	Invitrogen	Cat#4390843
Human LDLR siRNA-1<br>Forward: GCGACAGAUGCGAAAGAAATT<br>Reverse: UUUCUUUCGCAUCUGUCGCTT	This paper	In this study
Human LDLR siRNA-2<br>Forward: ACGGGAAAUGCAUCUCCUATT<br>Reverse: UAGGAGAUGCAUUUCCCGUTT	This paper	In this study
Human LDLR siRNA-3<br>Forward: GCUCUGAUGAGUCCCAGGATT<br>Reverse: UCCUGGGACUCAUCAGAGCTT	This paper	In this study
