# Supplementary material for: METTL3 drives NAFLD-related hepatocellular carcinoma and is a therapeutic target for boosting immunotherapy
Source: Cell Rep Med. 2023 Aug 15;4(8):101144. doi: 10.1016/j.xcrm.2023.101144 (PMC10439254; doi:10.1016/j.xcrm.2023.101144)
Supplement: Document S2. Article plus supplemental information [file mmc2.pdf]

Cell Reports Medicine

Article

METTL3 drives NAFLD-related hepatocellular carcinoma and is a therapeutic target for boosting immunotherapy

Graphical abstract

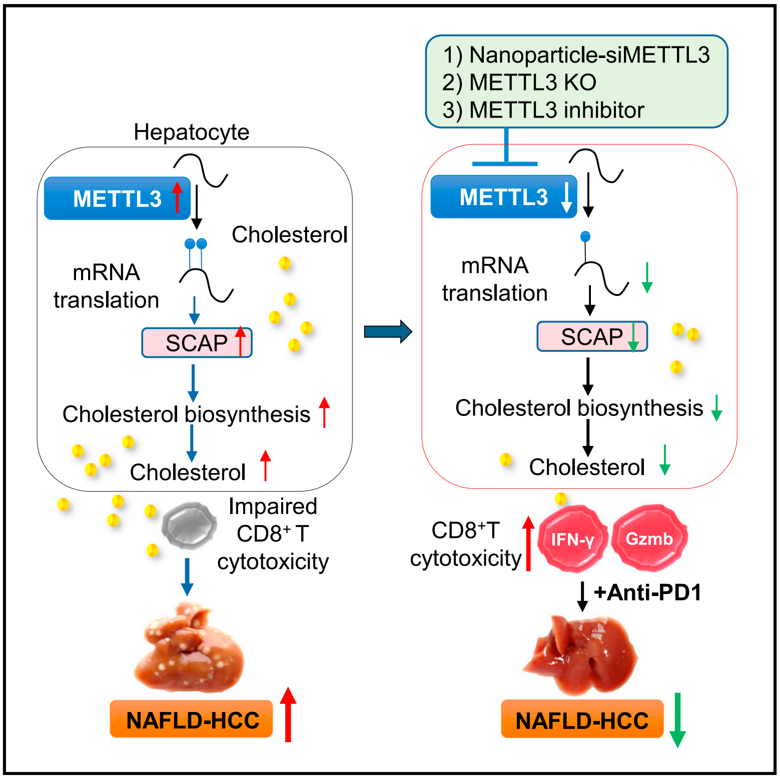

Highlights

- Liver-specific METTL3 knockin in mice promotes NAFLD-HCC development
- Tumor-intrinsic METTL3-mediated m^{6}A increases SCAP translation efficiency
- METTL3-SCAP axis promotes cholesterol accumulation and immunosuppression
- Inhibiting METTL3 plus PD-1 blockade improves response to immunotherapy

Authors

Yasi Pan, Huarong Chen, Xiang Zhang, ..., Alvin Ho-Kwan Cheung, Chi Chun Wong, Jun Yu

Correspondence

junyu@cuhk.edu.hk

In brief

Pan et al. identify that targeting METTL3 by small-molecule inhibitor STM2457 or nanoparticle siMETTL3 can induce a robust antitumor immune response, leading to the regression of NAFLD-HCC tumors when used in conjunction with anti-PD-1 therapy.

Pan et al., 2023, Cell Reports Medicine *4*, 101144
August 15, 2023 © 2023 The Author(s).
https://doi.org/10.1016/j.xcrm.2023.101144

CellPress

Cell Reports Medicine

CellPress OPEN ACCESS

Article

METTL3 drives NAFLD-related hepatocellular carcinoma and is a therapeutic target for boosting immunotherapy

Yasi Pan,[1] Huarong Chen,[1,2] Xiang Zhang,[1] Weixin Liu,[1] Yanqiang Ding,[1] Dan Huang,[2] Jianning Zhai,[1] Wenchao Wei,[1] Jun Wen,[1] Danyu Chen,[1] Yunfei Zhou,[1] Cong Liang,[1] Nathalie Wong,[5] Kwan Man,[3] Alvin Ho-Kwan Cheung,[4] Chi Chun Wong,[1] and Jun Yu[1,6,*]

[1]Institute of Digestive Disease and Department of Medicine and Therapeutics, State Key Laboratory of Digestive Disease, Li Ka Shing Institute of Health Sciences, The Chinese University of Hong Kong, Hong Kong SAR, China

[2]Department of Anaesthesia and Intensive Care, The Chinese University of Hong Kong, Hong Kong SAR, China

[3]Department of Surgery, Li Ka Shing Faculty of Medicine, The University of Hong Kong, Hong Kong SAR, China

[4]Department of Anatomical and Cellular Pathology, The Chinese University of Hong Kong, Hong Kong SAR, China

[5]Department of Surgery, The Chinese University of Hong Kong, Hong Kong SAR, China

[6]Lead contact

*Correspondence: junyu@cuhk.edu.hk

https://doi.org/10.1016/j.xcrm.2023.101144

SUMMARY

Non-alcoholic fatty liver disease (NAFLD) is an emerging risk factor of hepatocellular carcinoma (HCC). However, the mechanism and target therapy of NAFLD-HCC are still unclear. Here, we identify that the N^6-methyladenosine (m^6A) methyltransferase METTL3 promotes NAFLD-HCC. Hepatocyte-specific *Mettl3* knockin exacerbated NAFLD-HCC formation, while *Mettl3* knockout exerted the opposite effect in mice. Single-cell RNA sequencing revealed that METTL3 suppressed antitumor immune response by reducing granzyme B ($GZMB^+$) and interferon gamma-positive (IFN-γ^+) $CD8^+$ T cell infiltration, thereby facilitating immune escape. Mechanistically, METTL3 mediates sterol regulatory element-binding protein (SREBP) cleavage-activating protein (SCAP) mRNA m^6A to promote its translation, leading to the activation of cholesterol biosynthesis. This enhanced secretion of cholesterol and cholesteryl esters that impair $CD8^+$ T cell function in the tumor microenvironment. Targeting METTL3 by single-guide RNA, nanoparticle small interfering RNA (siRNA), or pharmacological inhibitor (STM2457) in combination with anti-programmed cell death protein 1 (PD-1) synergized to reinvigorate cytotoxic $CD8^+$ T cells and mediate tumor regression. Together, METTL3 is a therapeutic target in NAFLD-HCC, especially in conjunction with immune checkpoint blockade (ICB) therapy.

INTRODUCTION

Non-alcoholic fatty liver disease (NAFLD) refers to a spectrum of liver disorders defined by an ectopic fat deposition in the liver that is not due to alcohol consumption. The incidence of NAFLD has been rapidly rising in many developed countries and NAFLD is fast becoming a significant cause of hepatocellular carcinoma (HCC).[1] Compared to virus-associated HCC, NAFLD-HCC exhibits features including metabolic reprogramming, hepatic lipid accumulation, and lipotoxicity-driven chronic inflammation, which affect the therapeutic responsiveness of NAFLD-HCC. Immune checkpoint blockade (ICB) therapy, such as anti-programmed cell death protein 1 (PD-1)/programmed death-ligand 1 (PD-L1) antibodies, has revolutionized the management of virus-associated HCC.[2] Paradoxically, ICB therapy in NALFD-HCC results in a shortened survival compared to those with other malignancies.[3] Concordantly, preclinical studies demonstrate that ICB therapy in mice exacerbated NAFLD-HCC progression by aberrant re-activation of tissue-resident, auto-aggressive $CD8^+TNF^+PD\text{-}1^+$ T cells that induce liver damage, cirrhosis, and finally tumorigenesis.[4] Thus, there is an urgent need to identify the factor contributing to the tumor immune microenvironment of NAFLD-HCC and that serves as a therapeutic target to boost ICB therapy.

Epitranscriptomics comprises a myriad of RNA modifications critical to the post-transcriptional modulation of protein translation by regulating RNA stability, splicing, transport, and translation. Disruption in the epitranscriptome is a hallmark of cancer cells. RNA N^6-methyladenosine (m^6A) is the most abundant RNA modification dynamically regulated by writers (METTL3/METTL14/WTAP), erasers (FTO/ALKBH5), and readers (YTHDF1-3/YTHDC1-2) that respectively catalyze m^6A methylation and demethylation, and recognize m^6A-modified RNA.[5,6] Recent work has unveiled that m^6A regulators play multi-faceted roles in cancer, including cell growth,[7] self-renewal/stemness,[8] and metastasis.[9] Besides tumor-intrinsic effects, m^6A modification has been shown to regulate antitumor immunity.[10] Nevertheless, whether and how m^6A modulates

This is an open access article under the CC BY license (http://creativecommons.org/licenses/by/4.0/).

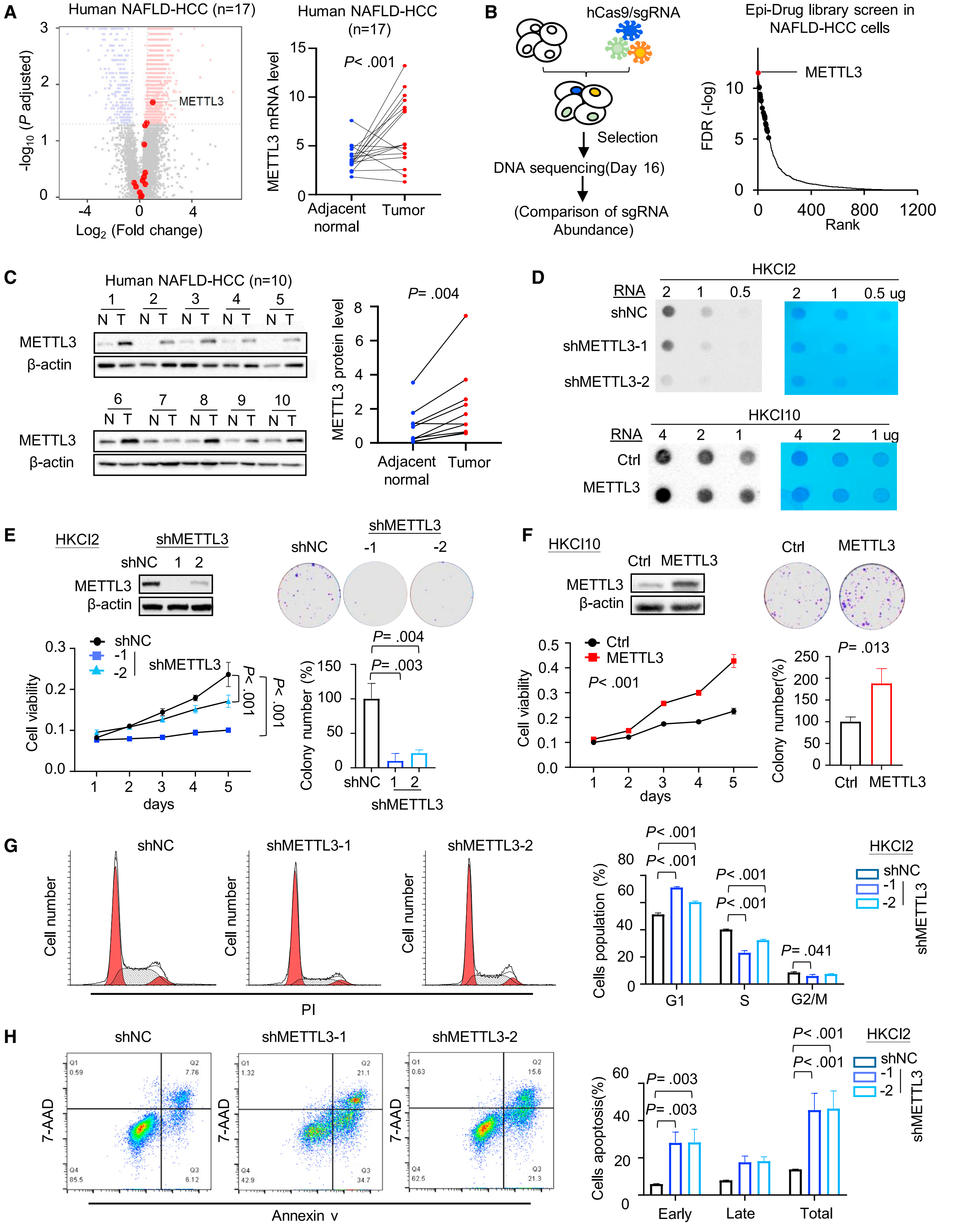

(legend on next page)

NAFLD-HCC is unknown. To identify therapeutic targets in NAFLD-HCC, we performed an integrated analysis of NAFLD-HCC transcriptomic sequencing[11] and gene essentiality datasets,[12] unraveling the m^{6}A writer METTL3 as a potential therapeutic target in NAFLD-HCC.

In this study, we investigated the oncogenic role of METTL3 in NAFLD-HCC using liver-specific *Mettl3* transgenic mice and *Mettl3* knockout mice and established an oncogenic effect of METTL3 in NAFLD-HCC progression. We deciphered the mechanism of action of METTL3, involving m^{6}A-modification of SREBP cleavage-activating protein (SCAP), which promotes cholesterol biosynthesis, subsequently impairing cytotoxic CD8$^+$ T cells in NAFLD-HCC. Targeting METTL3 reactivates CD8$^+$ T cell-mediated antitumor response and reverses anti-PD-1 resistance in NAFLD-HCC. METTL3 is thus a therapeutic target to improve anti-PD-1 treatment outcomes in NAFLD-HCC.

RESULTS

Integrative transcriptome and CRISPR-Cas9 screening identify METTL3 as an essential gene in NAFLD-HCC

We screened for druggable candidate genes in NAFLD-HCC based on two criteria: (1) overexpressed in NAFLD-HCC, and (2) essential for NAFLD-HCC cell survival. To this end, we inquired our in-house RNA sequencing (RNA-seq) database of 17 paired NAFLD-HCC and adjacent non-tumor tissues, revealing 2,028 genes that were overexpressed in NAFLD-HCC (fold change >1.5; $p < 0.005$), and METTL3 was one of the top RNA modification genes among the gene candidates (Figure 1A). Next, we performed CRISPR-Cas9 dropout screens in NAFLD-HCC cells to identify essential genes in NAFLD-HCC (Figure 1B). Our Epi-Drug library consists of 1,332 druggable genes from the human genome.[12] A total of 76 essential genes were identified to be critical for NAFLD-HCC cell survival (fold change < −1.5; false discovery rate [FDR] < 0.05) (Figures 1B and S1A). Overlap of these datasets narrowed to 22 common gene candidates (Figure S1A), of which METTL3 is the top-ranked candidate (Figure 1B). METTL3 is the primary m^{6}A methyltransferase that catalyzes m^{6}A modifications of mRNA. Among all m^{6}A regulators, only METTL3 was significantly upregulated in NAFLD-HCC (Figure S1B). Consistently, higher METTL3 expression at protein levels was observed in human NAFLD-HCC tissues compared to adjacent normal tissues (Figure 1C), as well as in mouse NAFLD-HCC tissues as compared to normal mouse liver tissues (Figure S1C).

We next validated the function of METTL3 *in vitro*. We performed METTL3 knockdown in HKCI2 cells expressing high endogenous METTL3, while METTL3 overexpression was done in HKCI10 cells with low basal METTL3 (Figure S1D). In line with the m^{6}A writer function of METTL3, dot blot demonstrated that METTL3 knockdown decreased RNA m^{6}A in human NAFLD-HCC cell line HKCI2 cells; conversely, ectopic expression of METTL3 increased m^{6}A abundance in NAFLD-HCC cell line HKCI10 cells (Figures 1D and S1E). METTL3 knockdown in HKCI2 cells inhibited cell growth (Figure 1E), induced G1 phase cell-cycle arrest (Figure 1G), and increased apoptosis (Figure 1H). Conversely, METTL3 overexpression exerted opposite effects in HKCI10 cells (Figures 1F and S1F). We thus identified METTL3 as an essential gene that is overexpressed in NAFLD-HCC.

Hepatocyte-specific METTL3 knockin exacerbates NAFLD-HCC in mice

To characterize the contribution of METTL3 to NAFLD-HCC, we constructed conditional *Mettl3* knockin mice by inserting *Mettl3* full-length coding sequence (CDS) into Rosa26 locus (*Rosa26-lsl-Mettl3*) (Figure 2A). Crossing *Rosa26-lsl-Mettl3* mice to *Albumin-Cre* mice resulted in hepatocyte-specific *Mettl3* knockin mice (*Mettl3^{LKI}*) (Figures 2A and S2A). *Mettl3^{LKI}* mice and their wild-type littermates were subjected to diethylnitrosamine (DEN) injection plus a choline-deficient, high-fat diet (CDHFD) to induce NAFLD-HCC (Figure 2B). Prior to sacrifice, we performed magnetic resonance imaging (MRI) (Figure 2C) and serum α-fetoprotein (AFP, a marker for liver cancer) detection (Figure 2D), both of which inferred accelerated liver tumorigenesis in *Mettl3^{LKI}* mice. Besides, significantly increased serum cholesterol, triglyceride (TG), and aspartate aminotransferase (AST) were observed in *Mettl3^{LKI}* mice (Figure 2E), implying exacerbated hyperlipidemia and liver damage. At sacrifice, we observed a significantly increased liver tumor number (p = 0.006) and tumor size ($p < 0.001$) in *Mettl3^{LKI}* mice compared to wild-type mice (Figures 2F, S2B, and S2C). Histological assessment (H&E) confirmed increased NAFLD-HCC formation in *Mettl3^{LKI}* mice (Figure 2F). We next performed Ki-67 staining to assess cell proliferation. Compared to wild-type mice, *Mettl3^{LKI}* mice demonstrated increased cell proliferation (Figure 2G). To independently verify the oncogenic function of METTL3 in NAFLD-HCC, we subjected *Mettl3^{LKI}* mice and wild-type littermates to DEN plus high-fat, high-cholesterol diet (HFHCD) (Figure 2H). Consistent findings were obtained,

Figure 1. METTL3 is overexpressed in NAFLD-HCC and is essential for NAFLD-HCC cell survival

(A) Volcano plot showing differentially expressed genes in 17 paired NAFLD-HCC and adjacent normal tissues by RNA-seq. RNA modification genes are marked as red circles (left). METTL3 expression was significantly induced in NAFLD-HCC (right).

(B) Workflow of CRISPR-Cas9 screening (left). METTL3 is a top candidate in NAFLD-HCC cells (HKCI2) by Epi-Drug library CRISPR-Cas9 screening (right).

(C) Protein expression of METTL3 in human NAFLD-HCC was validated in an independent cohort (n = 10).

(D) Dot blot quantification of m^{6}A abundance in mRNA transcripts in HKCI2 cells expressing shMETTL3 or shNC and in HKCI10 cells overexpressing METTL3. Methylene blue (MB) was used as a loading control. ShNC, shControl; shMETTL3, METTL3 knockdown.

(E) METTL3 knockdown in HKCI2 cells was confirmed by western blot. METTL3 knockdown suppressed cell proliferation and colony formation.

(F) METTL3 overexpression in HKCI10 cells was confirmed by western blot. METTL3 overexpression promoted HKCI10 cell growth and colony formation.

(G) METTL3 knockdown induced G1 phase cell-cycle arrest, as determined by propidium iodide (PI) staining and flow cytometry.

(H) METTL3 knockdown induced apoptosis as determined by annexin V-phycoerythrin/7-AAD staining and flow cytometry.

Data are presented as mean ± SD. Each spot represents one subject. Statistical significance was determined by one-way ANOVA or two-sided Student's t test where appropriate. 7-AAD, 7-aminoactinomycin D. See also Figure S1.

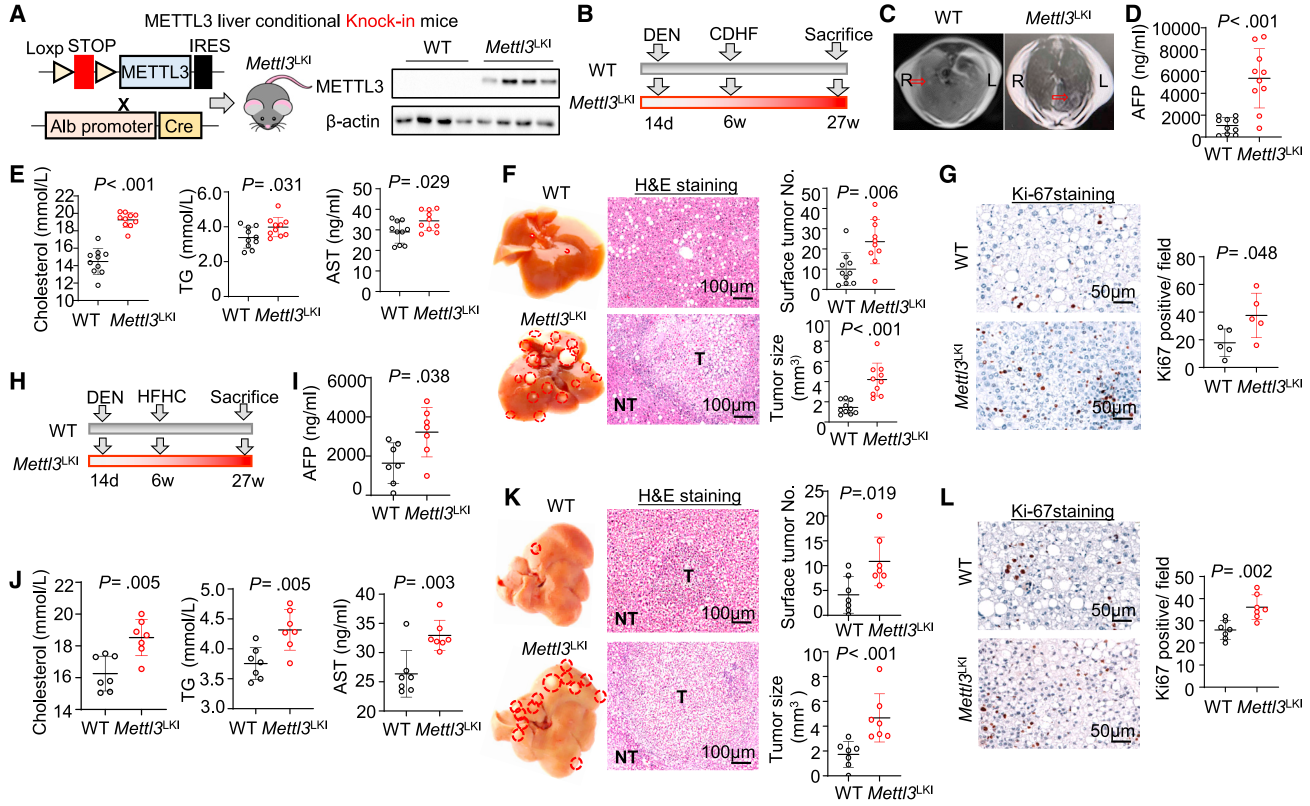

Figure 2. Hepatocyte-specific METTL3 knockin accelerates diet-induced NAFLD-HCC

(A) Design of hepatocyte-specific Mettl3 knockin mice (*Mettl3*^{LKI}). Western blot confirmed overexpression of METTL3 in the livers of *Mettl3*^{LKI} mice.

(B) Experimental design for diethylnitrosamine (DEN)-injected and CDHFD-induced NAFLD-HCC. At the age of 14 days, wild-type (WT) or *Mettl3*^{LKI} mice were injected with a single dose of DEN (5 mg/kg). Starting at 6 weeks of age, mice were fed with CDHFD until week 27.

(C) MRI for NAFLD-HCC tumors in WT and *Mettl3*^{LKI} mice.

(D) Serum AFP levels in WT and *Mettl3*^{LKI} mice.

(E) Hepatocyte-specific METTL3 overexpression increased serum cholesterol, TG, and AST levels.

(F) Representative gross morphology, H&E staining, surface tumor number, and tumor size in CDHFD-induced NAFLD-HCC in WT and *Mettl3*^{LKI} mice (n = 10/group).

(G) Ki-67 staining of livers of WT and *Mettl3*^{LKI} mice.

(H) Experimental design for DEN-injected and HFHCD-induced NAFLD-HCC. At the age of 14 days, WT or *Mettl3*^{LKI} mice were injected with a single dose of DEN (5 mg/kg). Starting at 6 weeks of age, mice were fed with HFHCD until week 27.

(I) Hepatocyte-specific *Mettl3*^{LKI} increased serum AFP.

(J) Serum cholesterol, TG, and AST levels in WT and *Mettl3*^{LKI} mice treated with HFHCD.

(K) *Mettl3*^{LKI} promoted HFHCD-induced NAFLD-HCC, as shown by tumor number, tumor size, and H&E staining (n = 7/group).

(L) Ki-67 staining in the livers of WT and *Mettl3*^{LKI} mice.

Data are presented as mean ± SD. Each spot represents one subject. Statistical significance was determined by one-way ANOVA or two-sided Student's t test where appropriate. AFP, α-fetoprotein; AST, aspartate aminotransferase; CDHFD, choline-deficient high-fat diet; H&E, hematoxylin and eosin; HFHCD, high-fat, high-cholesterol diet; TG, triglyceride. See also Figure S2.

showing that *Mettl3*^{LKI} mice had higher serum AFP (Figure 2I), increased serum cholesterol, triglycerides, and AST levels (Figure 2J) and developed more liver tumors with larger size (Figures 2K and S2D). Ki-67 staining revealed a consistent increase in cell proliferation in liver tumors of *Mettl3*^{LKI} mice (Figure 2L). Meanwhile, *Mettl3*^{LKI} mice and their wild-type counterparts given a normal chow diet demonstrated no difference in liver histology and liver function markers (AST, alanine aminotransferase [ALT], TG, and cholesterol) (Figures S2E–S2G). Collectively, these results demonstrate that hepatocyte-specific *Mettl3* knockin in mice accelerates diet-induced NAFLD-HCC by promoting hyperlipidemia and cell proliferation.

METTL3 knockout suppresses NAFLD-HCC formation in mice

To validate the function of METTL3 in NAFLD-HCC, we next established *Mettl3* heterozygous knockout (*Mettl3*$^{+/-}$) mice (Figure 3A), which did not affect CD8^{+} T cell proliferation and function (Figures S3A–S3E). *Mettl3*$^{+/-}$ mice and wild-type littermates were given DEN plus HFHCD to induce NAFLD-HCC (Figure 3B). At 21 weeks after DEN injection, serum AFP was reduced in *Mettl3*$^{+/-}$ mice compared to wild-type mice (Figure 3C). Upon sacrifice, *Mettl3*$^{+/-}$ mice exhibited smaller liver tumor number (p = 0.048) and size (p = 0.010) compared to wild-type mice (Figures 3D and S3F). The serum cholesterol

CellPress
OPEN ACCESS

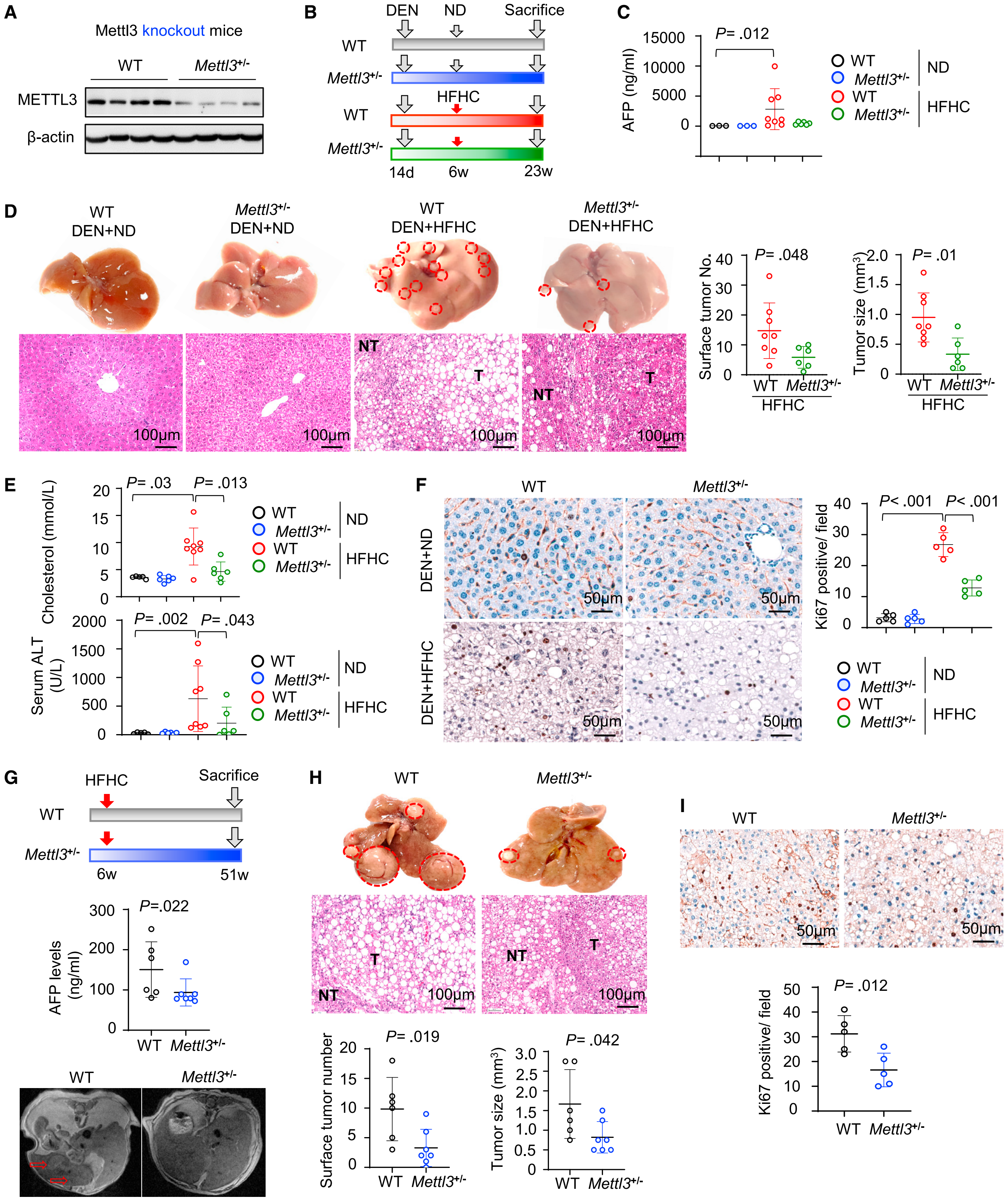

Figure 3. METTL3 knockout suppresses diet-induced NAFLD-HCC

(A) Western blot confirmed METTL3 knockout in the livers of *Mettl3*$^{+/-}$ mice.

(B) Experimental design for a single dose of DEN (25 mg/kg) at day 14 plus normal diet (ND) as the control group, or DEN (25 mg/kg) plus HFHCD starting at week 6 to induce NAFLD-HCC.

(legend continued on next page)

(p = 0.013) and ALT (p = 0.043) were reduced in HFHCD-treated *Mettl3*$^{+/-}$ mice compared to wild-type controls (Figure 3E). DEN plus HFHCD increased cell proliferation in the livers of wild-type mice, which was markedly attenuated in *Mettl3*$^{+/-}$ mice (p < 0.001) (Figure 3F). Moreover, we established a spontaneous model of NAFLD-HCC by feeding mice with HFHCD for 45 weeks (Figure 3G).[13] Consistently, METTL3 knockout inhibited NAFLD-HCC development, as shown by reduced AFP and MRI measurements (Figure 3G) and the reduced tumor number (p = 0.019) and size (p = 0.042) at sacrifice (Figures 3H and S3G). Concordantly, cell proliferation was also reduced in *Mettl3*$^{+/-}$ mice (Figure 3I). Our data imply that METTL3 depletion could impair NAFLD-HCC development and is a potential therapeutic target.

Hepatocyte-specific METTL3 knockin impairs antitumor immunity in NAFLD-HCC

To decipher the molecular mechanism of METTL3-induced NAFLD-HCC, we performed RNA-seq of NAFLD-HCC tumor tissues from *Mettl3*^{LKI} and wild-type mice. Notably, immune-related pathways, such as cytokine-cytokine receptor interaction, primary immunodeficiency, and chemokine signaling pathways, were significantly downregulated in *Mettl3*^{LKI} tumors by gene set enrichment analysis (GSEA) (Figures 4A and S4A), implying that hepatocyte-specific METTL3 knockin reshapes immune microenvironment in NAFLD-HCC. Against this backdrop, we performed single-cell RNA-seq (scRNA-seq) of CD45^{+} cell populations from liver tumors of *Mettl3*^{LKI} mice and wild-type controls (Figure 4B). Non-supervised t-distributed stochastic neighbor embedding (t-SNE) identified distinct immune cell clusters, including B cells (Cd19^{+}Cd79^{+}), dendritic cells (Cdllc^{+}Lv6c2^{+}), Kupffer cells (F4/80^{+}Csflr^{+}), macrophages (Mrcl^{+}), myeloid-derived suppressor cells (MDSC) (Ly6g^{+} S100a8^{+}S100a9^{+}), natural killer (NK)/natural killer T cells (NKT) (Klrblc^{+}Ncrl^{+}), and T cells (Klrblc^{-}Trbcl^{+}) (Figure 4C). Among these, the T cells cluster was significantly depleted (p < 0.001) in *Mettl3*^{LKI} mice, together with the downregulation of NK cells, whereas Kupffer cells and B cells showed an increasing trend (Figure 4C). This implies that hepatocyte-specific METTL3 might regulate T cell activity in NAFLD-HCC. We next divided the T cells and NK cells population into eight clusters (Figures 4D, 4E, and S4B). Notably, infiltration of GZMB^{+} and interferon gamma-positive (IFN-γ^{+}) CD8^{+} T cells (clusters 0 and 4) was reduced in *Mettl3*^{LKI} mice, suggesting hepatocyte-specific *Mettl3* knockin inhibited cytotoxic CD8^{+} T activation in NAFLD-HCC (Figures 4F and 4G).

To understand the molecular mechanisms that attenuate CD8^{+} T cells effector phenotype acquisition, we also performed GSEA to query the Hallmark database. Tumor-intrinsic METTL3 knockin significantly enhanced the expression of crucial pathways for CD8^{+} T cell dysfunction, such as cholesterol metabolism, ferroptosis, phagosome, and lysosome (Figure 4H). In agreement with this, flow cytometry showed that *Mettl3*^{LKI} mice had reduced total T cells (CD3^{+}) (Figure 4I) and expression of activation markers IFN-γ and GZMB in both CD3^{+} and CD8^{+} cell subsets (Figure 4J). Corroborating with impaired T cell function, serum IFN-γ levels were reduced in *Mettl3*^{LKI} mice compared to control mice (Figure 4K). In addition, CD8^{+}PD-1^{+} T cells were upregulated in our *Mettl3*^{LKI} mouse tumor, implying that METTL3 suppressed T cell effector function and promoted T cell exhaustion (Figures S4C and S4D). Other immune cell types, such as CD4^{+} T cells, regulatory T cells (Tregs), or NK cells, showed no significant differences (Figures S4E–S4G). All these data led us to ask if METTL3-mediated NAFLD-HCC depends on its effect on CD8^{+} T cells. To this end, we performed CD8^{+} T cell depletion in orthotopic NAFLD-HCC models (Figures 4L, S4H, and S4I). Hepal-6-Luc cells expressing control (sgNC) or METTL3 knockout (sgMETTL3) were implanted into the nonalcoholic steatohepatitis (NASH) livers of C57BL/6 mice, followed by treatment with anti-CD8α or immunoglobulin (Ig) G control. As shown in Figure 4M, METTL3 depletion significantly inhibited orthotopic NAFLD-HCC growth in IgG control, whereas such an inhibitory effect was abolished after the depletion of CD8^{+} T cells by anti-CD8α. Our results indicated that tumor-intrinsic METTL3 promotes NAFLD-HCC by attenuating cytotoxic CD8^{+} T cell response.

METTL3 promotes cholesterol biosynthesis in NAFLD-HCC cells

To gain insight into molecular events perturbed by METTL3 in NAFLD-HCC cells, we performed integrative RNA-seq and m^{6}A sequencing (m^{6}A-seq) in HKCI2 cells with or without METTL3 knockdown. A total of 149 genes were downregulated, while 192 genes were upregulated (|fold change| > 2, adjusted p < 0.05) in METTL3 knockdown cells (Figure S5A). Pathway enrichment of differentially expressed genes revealed cholesterol biosynthesis as the top enriched pathway (Figure 5A). Volcano plot revealed the downregulation of SREBP2, a master transcription factor for cholesterol biosynthesis in METTL3 knockdown cells, together with enzymes in the mevalonate pathway (HMGCS1, HMGCR, MVK, FDFTI, and SQLE) and distal cholesterol biosynthesis (LSS, DHCR7, and DHCR24) (Figures 5B, left, and S5B). GSEA also showed significant depletion of cholesterol biosynthesis in METTL3 knockdown cells (Figure 5B, right). Western blot validated that METTL3 knockdown in HKCI2 cells suppressed protein expression of HMGCR, SQLE, and

(C) Serum AFP levels in WT or *Mettl3*$^{+/-}$ mice fed with ND or HFHCD.
(D) Representative gross morphology, H&E staining, tumor number, and tumor size in livers of WT or *Mettl3*$^{+/-}$ mice (n = 6–8/group).
(E) METTL3 knockout suppressed serum cholesterol and ALT.
(F) Ki-67 staining of WT or *Mettl3*$^{+/-}$ mouse livers.
(G) Experimental design for HFHCD-induced spontaneous NAFLD-HCC. Tumor formation was monitored by MRI and serum AFP.
(H) METTL3 knockout reduced tumor formation as determined by H&E staining, tumor number, and tumor size (n = 6–7/group).
(I) Ki-67 staining of livers of HFHCD fed WT and *Mettl3*$^{+/-}$ mice.
Data are presented as mean ± SD. Each spot represents one subject. Statistical significance was determined by one-way ANOVA or two-sided Student's t test where appropriate. ALT, alanine aminotransferase. See also Figure S3.

CellPress
OPEN ACCESS

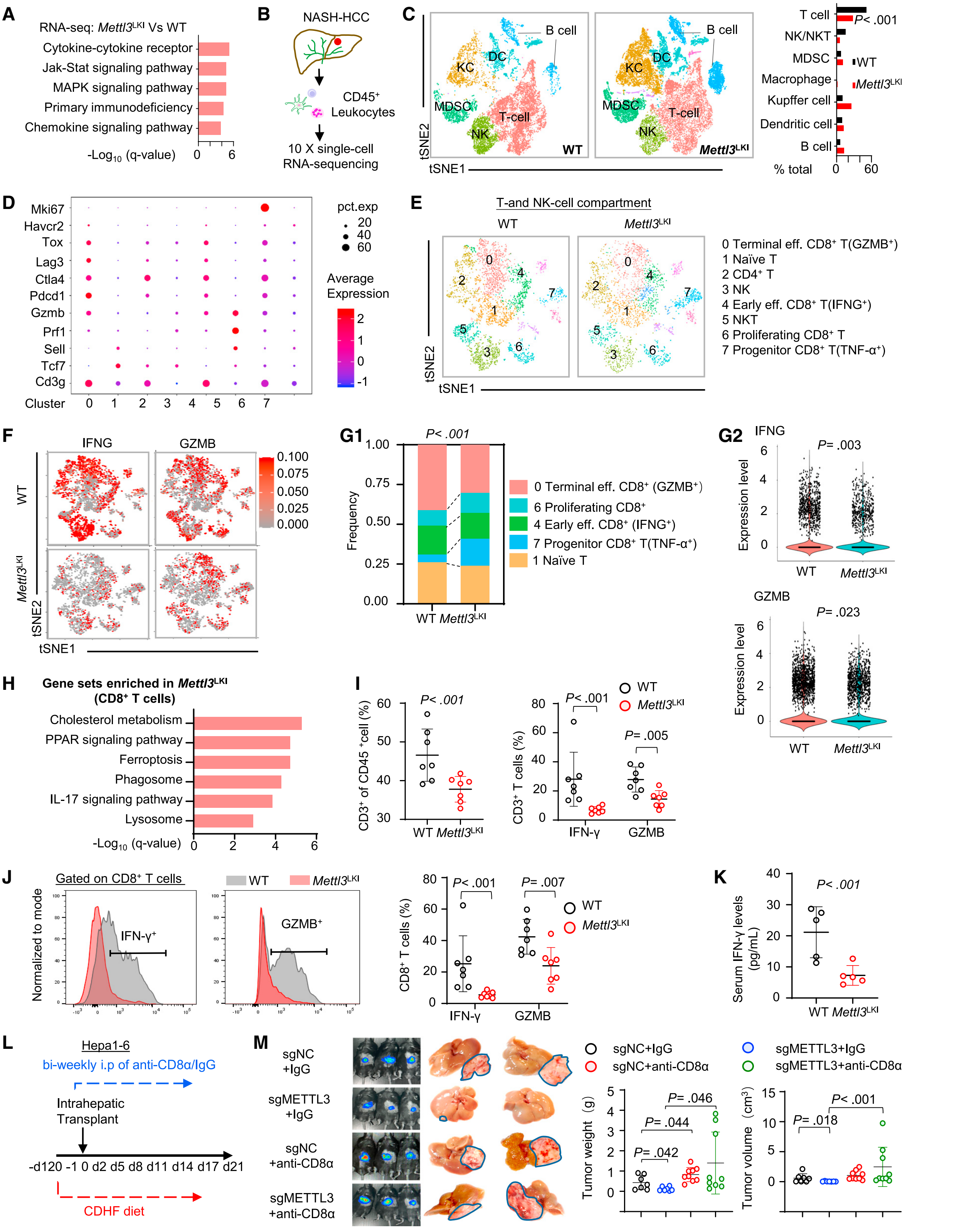

(legend on next page)

SREBP2, key rate-limiting enzymes in cholesterol biosynthesis (Figures 5C and S5C). Accordingly, free cholesterol and cholesteryl esters were reduced by shMETTL3 in HKCI2 cells (Figure 5C). Conversely, METTL3 overexpression in HKCI10 cells promoted HMGCR, SQLE, and SREBP2 expression and induced cellular cholesterol levels (Figures 5D and S5C). In line with *in vitro* findings, hepatic cholesterol and cholesteryl esters were increased in *Mettl3*^{LKI} mice (Figure 5E), whereas their levels were downregulated in *Mettl3*$^{+/-}$ mice (Figure 5F). Corroborating these data, METTL3 mRNA expression positively correlated with HMGCR, SQLE, and SREBP2 mRNA in our human NAFLD-HCC cohort (Figures 5G and S5D). Hence, our data indicated that METTL3 might drive aberrant cholesterol biosynthesis in NAFLD-HCC.

SCAP is a critical target of METTL3 in promoting cholesterol biosynthesis

We next asked if METTL3-mediated m^{6}A underlies positive regulation of the cholesterol biosynthesis pathway by performing m^{6}A-seq in HKCI2 cells with METTL3 knockdown (Figure S5E). METTL3 depletion downregulated m^{6}A peaks primarily in the CDS and 3′ UTR (Figure 5H). In total, the knockdown of METTL3 significantly reduced the number of m^{6}A peaks (N = 5,563) as compared to the control group (N = 8,894) (Figure S5F). We overlapped the potential m^{6}A targets of METTL3 with cholesterol biosynthesis pathway, unveiling one common gene, SCAP (Figure 5I). SCAP has been shown to promote cholesterol biosynthesis by promoting proteolytic activation of SREBP2 in Golgi.[14] UCSC snapshots of m^{6}A-seq reads indicated knockdown of METTL3-repressed m^{6}A modification of SCAP mRNA (Figure 5J), which was confirmed by methylated RNA immunoprecipitation (MeRIP)-qPCR (Figures 5J and S5G). Conversely, METTL3 overexpression increased m^{6}A modification of SCAP (Figure 5J). Moreover, ribosome sequencing (Ribo-seq) and ribosome nascent-chain complex-bound mRNA-qPCR (RNC-qPCR) both showed that METTL3-mediated m^{6}A increased SCAP translation (Figure 5K). To solidify the direct effect of SCAP mediated by METTL3, RNA immunoprecipitation (RIP)-qPCR assay with anti-METTL3 antibody was performed. We found that METTL3 pull-down led to the enrichment of SCAP mRNA compared to IgG control (Figure 5L). Consistently, METTL3 knockdown abrogated enrichment of SCAP mRNA (Figure 5L), confirming its direct binding to SCAP mRNA. We then generated a luciferase reporter by inserting the SCAP 3′ UTR downstream of firefly luciferase gene. Either shMETTL3 or METTL3 inhibitor STM2457 significantly reduced SCAP 3′ UTR luciferase reporter activity (Figure 5M), suggesting that METTL3 inhibition suppressed SCAP translation. In support of this, shMETTL3 inhibited SCAP protein levels in HKCI2 cells, and METTL3 overexpression exerted an opposite effect in HKCI10 cells (Figure 5N) while not affecting SCAP mRNA (Figure S5H). Notably, catalytically inactive mutant METTL3 failed to promote SCAP protein expression (Figure 5O), implying that METTL3 promotes SCAP translation in an m^{6}A-dependent manner. Consistent with *in vitro* observations, SCAP protein, but not its mRNA, was upregulated in tumors from *Mettl3*^{LKI} mice compared to those from control mice (Figures 5P and S5I). Furthermore, a positive correlation between SCAP and METTL3 protein expression was found in paired NAFLD-HCC tissues (N = 10, $p < 0.001$) (Figures 5Q and S5J). To identify m^{6}A readers involved in METTL3-induced SCAP translation, we performed knockdown of m^{6}A readers (YTHDF1/2/3, YTHDC1/2, and IG2BP1/2/3) in NAFLD-HCC cells overexpressing METTL3 (Figure S5K). Among m^{6}A readers, YTHDC1 knockdown significantly suppressed SCAP protein expression (Figure S5K). Consistently, siYTHDC1 reduced SCAP 3′ UTR reporter activity (Figure S5L). RIP-qPCR assay confirmed the direct binding of YTHDC1 to SCAP mRNA, which was enhanced upon METTL3 overexpression (Figure S5M). We next asked whether SCAP is involved in METTL3-mediated cholesterol biosynthesis. Indeed, siSCAP abolished the induction of cholesterol by METTL3 overexpression in HKCI10 cells (Figure 5R). siSCAP or siMETTL3 reduced the expression of cholesterol biosynthesis genes and extracellular cholesterol levels (Figures S5N and S5O). Together, our results suggested SCAP as a downstream target of METTL3 in promoting cholesterol biosynthesis in NAFLD-HCC.

Tumor-intrinsic METTL3 impairs CD8$^+$ T cell function via cholesterol biosynthesis

Cholesterol has been reported to induce CD8$^+$ T cell exhaustion in tumor microenvironment.[15] We thus asked if METTL3-induced cholesterol biosynthesis modulated CD8$^+$ T cell function in

Figure 4. NAFLD-HCC tumor-intrinsic METTL3 restricts the activation and effector status of CD8$^+$ T cells

(A) Immune-response-related pathways were downregulated in NAFLD-HCC from *Mettl3*^{LKI} mice by RNA-seq.

(B) Schematic diagram depicting single-cell RNA-seq and downstream analysis.

(C) Identification of tumor-infiltrating immune cell populations. Single-cell RNA sequencing (scRNA-seq) of tumor-infiltrating immune cells showed that T cells were depleted in *Mettl3*^{LKI} mice. Representative of one experiment; n = 5 pooled WT mice and n = 5 pooled *Mettl3*^{LKI} mice.

(D) Gene markers for different clusters of tumor-infiltrating T/NK-cell populations.

(E) Identification of tumor-infiltrating T/NK-cell populations.

(F) Distribution and expression of IFN-γ and GZMB among individual tumor-infiltrating T/NK-cells.

(G) Proportion of different clusters of CD8$^+$ T cells (G1), and violin plots showing the expression of IFN-γ and GZMB in CD8$^+$ T cells from WT and *Mettl3*^{LKI} (G2).

(H) GSEA of differentially expressed genes in CD8$^+$ T cells within tumors of *Mettl3*^{LKI} mice and WT mice.

(I) Validation of infiltration of CD3$^+$ T cells and their activation markers IFN-γ and GZMB expression in WT and *Mettl3*^{LKI} mouse tumors by flow cytometry.

(J) Infiltration of IFN-γ$^+$ and GZMB$^+$ CD8$^+$ T cells in WT and *Mettl3*^{LKI} mouse tumors by flow cytometry.

(K) Serum IFN-γ was decreased in *Mettl3*^{LKI} mice compared to WT mice.

(L) Schematic diagram for anti-CD8a depletion regime in the orthotopic NAFLD-HCC model.

(M) Representative images of livers at day 21 post intrahepatic injection for orthotopic NAFLD-HCC tumors with or without METTL3 and treated with IgG or anti-PD-1.Quantification of tumor weight and tumor volume in orthotopic NAFLD-HCC mice (n = 7–9/group). Data are presented as mean ± SD. Each spot represents one subject. Statistical significance was determined by one-way ANOVA or two-sided Student's t test where appropriate. See also Figure S4.

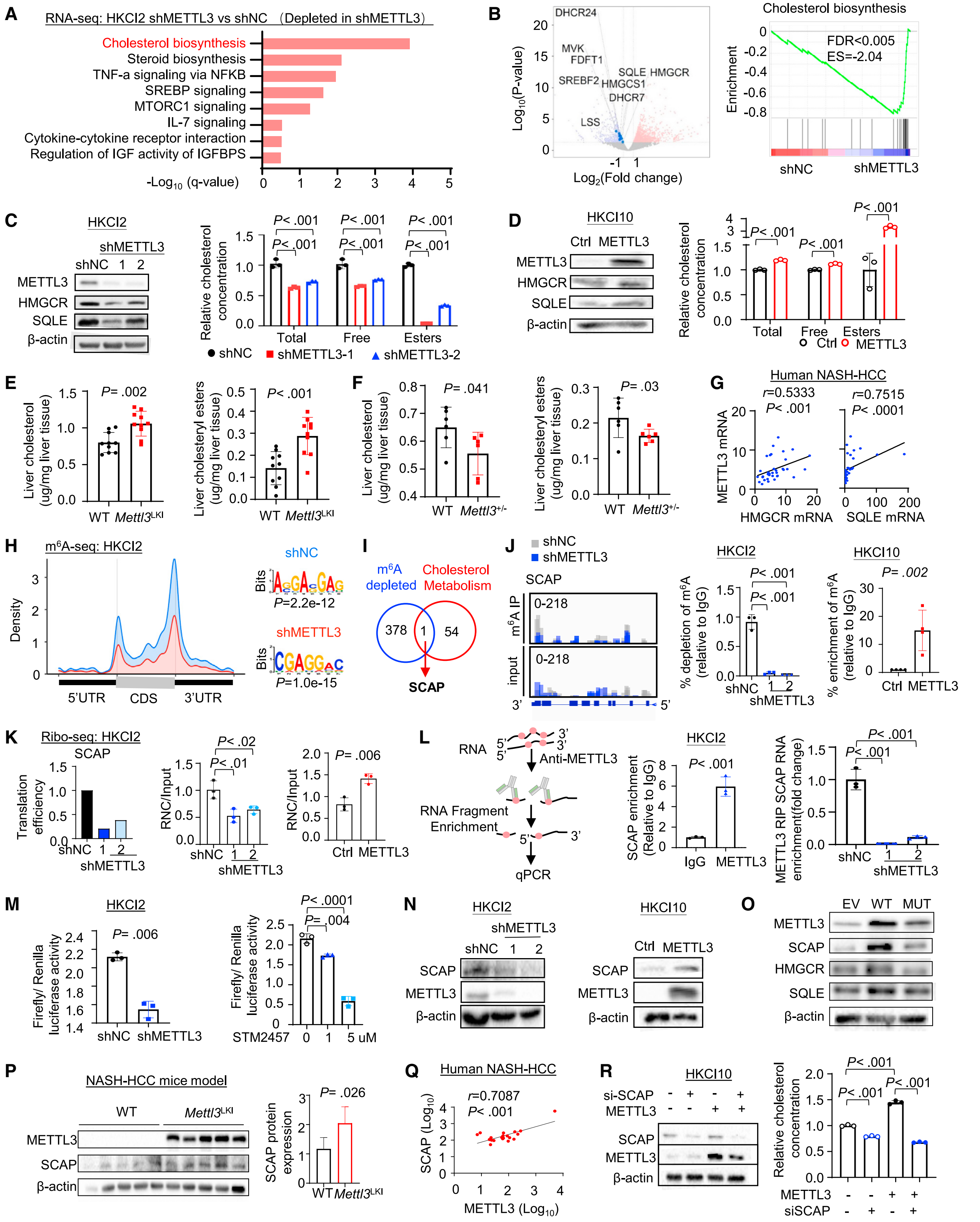

(legend on next page)

NAFLD-HCC. We isolated T cells from tumor-bearing mice and cultured T cells in a conditioned medium from murine Hepa1-6 cells with or without METTL3 knockout (Figure 6A). We found that conditioned medium from control Hepa1-6 significantly impaired CD8$^+$ T cells response compared to blank medium, as exemplified by reduced IFN-γ and granzyme B expression (Figure 6B). Notably, conditioned medium from METTL3 knockout cells largely reactivated CD8$^+$ T cells compared to that from control cells (Figure 6B). More importantly, conditioned medium from METTL3 knockdown in human NAFLD-HCC cells (HKCI2) boosted the antitumor response of human CD8$^+$ T cells (Figure 6C). The exogenous addition of cholesterol and cholesteryl esters abrogated CD8$^+$ T cell activation induced by shMETTL3 (Figure 6D). Conversely, overexpression of METTL3 in HKCI10 suppressed IFN-γ^+ and granzyme B$^+$ in CD8$^+$ T cells, an effect reversed by cholesterol sequestration using β-cyclodextrin (Figure 6E). The low-density lipoprotein receptor (LDLR) knockdown to suppress cholesterol uptake in T cells abolished the immunosuppressive effect mediated by METTL3-overexpression *in vitro* (Figures S6A and S6B). To verify the role of cholesterol in METTL3-induced NAFLD-HCC, we treated orthotopic NAFLD-HCC overexpressing METTL3 with simvastatin, a cholesterol biosynthesis inhibitor. Simvastatin significantly abolished the tumor-promoting effect of METTL3 (Figures S6C and S6D). Flow cytometry showed simvastatin reactivated IFN-γ^+ and GZMB$^+$ CD8$^+$ T cells in METTL3-overexpressing NAFLD-HCC allografts (Figure S6E). Thus, cholesterol inhibition mitigated METTL3-induced immunosuppression and tumor growth in mice. Corroborating these results, T cell-killing assays also showed that CD8$^+$ T cells harvested from NAFLD-HCC have tumor cell-killing ability (Figures 6F, 6G, and S6F), which was suppressed by cholesterol supplementation in a dose-dependent manner (Figure 6G). These findings thus underscore the role of cholesterol in METTL3-induced inactivation of antitumor CD8$^+$ T cells in NAFLD-HCC.

Given that SCAP is the downstream factor driving cholesterol biosynthesis, we asked if SCAP is involved in the inhibitory effect of METTL3 on CD8$^+$ T cell function. Consistent with our hypothesis, SCAP knockdown (siSCAP) in METTL3-overexpressing HKCI2 (Figure 6H) and HKCI10 (Figure 6I) cells reinvigorated CD8$^+$ T cell function, in line with reduced cholesterol levels (Figure 5R). We thus elucidated a METTL3-m^{6}A-SCAP-cholesterol axis that antagonizes CD8$^+$ T cell function in NAFLD-HCC to promote tumorigenesis.

Targeting of METTL3 synergizes with anti-PD-1 therapy to inhibit NAFLD-HCC

Since METTL3 inactivates CD8$^+$ T cells in NAFLD-HCC microenvironment, this prompted us to explore whether targeting METTL3 could improve ICB therapeutic efficacy for NAFLD-HCC. A syngeneic orthotopic NAFLD-HCC model was established by injecting Hepa1-6-Luc cells with or without METTL3 knockout into NASH livers of C57BL/6J mice, followed by treatment with anti-PD-1 or IgG isotype (Figure 7A). Remarkably, METTL3 plus anti-PD-1 therapy demonstrated synergistic inhibition of tumor growth as compared to control ($p = 0.008$), sgMETTL3 ($p = 0.032$), and anti-PD-1 ($p = 0.008$) groups (Figures 7B and 7C), achieving >90% reduction in tumor volume and weight. Moreover, the highest infiltration of IFN-γ^+ and GZMB$^+$ CD8$^+$ T cells was detected in the sgMETTL3 tumors treated with anti-PD-1 (Figure 7D). The synergistic antitumor effect of METTL3 knockout plus anti-PD-1 treatment was further verified in a second orthotopic NAFLD-HCC mouse model using RIL-175 cells (Figures S4A–S4C). These results indicated that targeting METTL3 potentiates anti-PD-1 therapy to suppress NAFLD-HCC.

We next explored the effectiveness of anti-METTL3 therapeutics in potentiating anti-PD-1 therapy in NAFLD-HCC. First, we utilized a nanoparticle-based small interfering RNA (siRNA) approach[16,17] to target METTL3. Pyrimidine bases (C/U) in

Figure 5. METTL3 promotes cholesterol biosynthesis via m^{6}A modification of SCAP
(A and B) RNA-seq showed that METTL3 knockdown in HKCI2 cells reduced cholesterol biosynthesis.
(C) HMGCR and SQLE proteins were suppressed by shMETTL3 in HKCI2 cells. (Left). Total free cholesterol and esters were decreased in HKCI2-shMETTL3 cells (right).
(D) METTL3 overexpression induced HMGCR and SQLE protein expression, as well as cholesterol and cholesteryl esters levels, in HKCI10 cells.
(E) Hepatic cholesterol and cholesteryl esters levels in WT and *Mettl3*^{LKI} mice with diet-induced NAFLD-HCC.
(F) Hepatic cholesterol and cholesteryl esters levels in WT and *Mettl3*$^{+/-}$ mice with diet-induced NAFLD-HCC.
(G) Correlation of METTL3 with cholesterol biosynthesis genes in human NAFLD-HCC.
(H) Overall m^{6}A profiles identified by m^{6}A-seq in HKCI2 cells with or without METTL3 knockdown. Normalized distribution of m^{6}A peaks and the identified m^{6}A motifs.
(I) Overlap m^{6}A-depleted peaks with cholesterol metabolism revealed SCAP as a METTL3 target.
(J) UCSC snapshots of the m^{6}A-seq reads of SCAP mRNA (left). Normalized read density levels are shown in gray (shNC) or blue (shMETTL3). MeRIP-qPCR of m^{6}A levels of SCAP mRNA in HKCI2 cells with METTL3 knockdown and HKCI10 cells with METTL3 overexpression.
(K) Ribo-seq showed repressed SCAP translation in METTL3 knockdown in HKCI2 (left). RNC-qPCR of SCAP in HKCI2 with METTL3 knockdown (middle) and HKCI10 cells with METTL3 overexpression (right).
(L) Schematic of METTL3-RIP-qPCR in HKCI2 cells (left). Relative enrichment of SCAP RNA by METTL3 in HKCI2 cells (middle). Relative enrichment of SCAP upon METTL3 knockdown in HKCI2 by METTL3-RIP-qPCR (right).
(M) Luciferase activity of SCAP 3′ UTR in HKCI2 cells upon METTL3 knockdown (left) or treated with METTL3 inhibitor STM2457 (right) relative to Renilla luciferase activity.
(N) SCAP protein expression in HKCI2 cells with METTL3 knockdown, and HKCI10 cells with METTL3 overexpression.
(O) Mutant METTL3 failed to promote protein expression of SCAP, HMGCR, and SQLE.
(P) SCAP protein expression in WT and *Mettl3*^{LKI} with diet-induced NAFLD-HCC.
(Q) Correlation of SCAP and METTL3 protein levels in human NAFLD-HCC, as determined by western blot and densitometry analysis.
(R) SCAP knockdown in HKCI10 cells was determined by western blot (left). siSCAP reversed METTL3-induced cholesterol levels (right).
Data are presented as mean ± SD. Each spot represents one subject. Statistical significance was determined by one-way ANOVA or two-sided Student's t test where appropriate. See also Figure S5.

CellPress
OPEN ACCESS

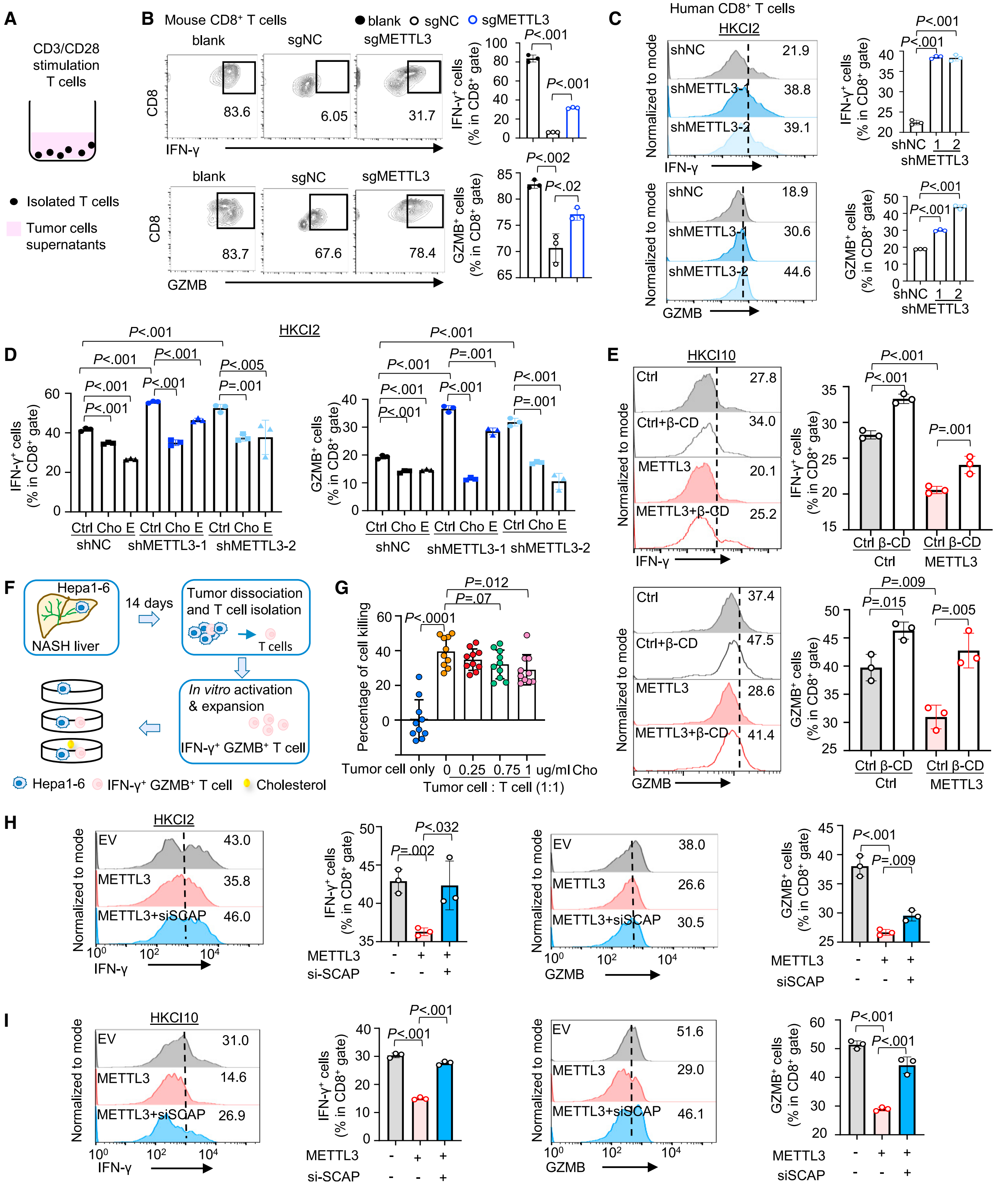

Figure 6. METTL3-mediated SCAP impairs CD8+ T cell function through cholesterol accumulation

(A) Design of T cell coculture with tumor cell conditioned medium. Naive T cells were *in vitro* stimulated with CD3/CD28 beads in the presence of interleukin (IL)-2 (10 ng/mL). CD8+ T cell IFN-γ and GZMB expression was examined after 72 h by flow cytometry.

(legend continued on next page)

siRNAs were modified with 2′-*O*-methylation to ensure RNA stability, and cyanine 5 fluorescent tag was incorporated to monitor siRNA delivery *in vivo* (Figures 7E and 7F). METTL3-siRNA efficiency was validated *in vitro* (Figure S7D). We established orthotopic NAFLD-HCC using Hepa1-6-Luc cells and monitored tumor growth by luciferase imaging. After tumor establishment (day 9), mice were randomized to receive nanoparticle-siNC or nanoparticle-siMETTL3, with or without anti-PD-1 treatment (Figure 7F). As expected, targeting of METTL3 plus anti-PD-1 exerted the strongest inhibitory effect on tumor growth *in vivo* (Figures 7G and 7H), concomitant with the highest level of intratumor IFN-γ^+ and GZMB$^+$ CD8$^+$ T cells (Figure 7I).

Finally, we investigated whether the pharmacological blockade is effective in potentiating anti-PD-1 therapy in NAFLD-HCC. STM2457 is a highly potent and selective METTL3 inhibitor.[18] We treated orthotopic NAFLD-HCC tumors (Hepa1-6) with STM2457, anti-PD-1, or their combination (Figure 7J). Single STM2457 significantly inhibited tumor growth ($p < 0.05$), while anti-PD-1 had no effect. Importantly, a combination of STM2457 and anti-PD-1 exerted an additive effect against tumor growth as compared to control or single treatments ($p < 0.05$) (Figures 7K and 7L). Furthermore, STM2457 plus anti-PD-1 treatment most strongly induced IFN-γ^+ and GZMB$^+$ CD8$^+$ T cells in NAFLD-HCC tumors (Figure 7M). Taken together, targeting METTL3 in combination with anti-PD-1 holds promise for the effective treatment of NAFLD-HCC.

DISCUSSION

In this study, RNA-seq unraveled that METTL3 is an m^{6}A regulator overexpressed in NAFLD-HCC compared to adjacent normal tissues. Underscoring the functional role of METTL3 in NAFLD-HCC, CRISPR-Cas9 dropout screening identified METTL3 as a top essential gene for NAFLD-HCC cell survival. Against this backdrop, we established transgenic mouse models with liver-specific, conditional METTL3 knockin or METTL3 knockout and tested its effect on tumorigenesis in two dietary models of NAFLD-HCC. Consistent with our hypothesis, liver-specific METTL3 knockin increased NAFLD-HCC incidence and tumor burden. Conversely, knockout of METTL3 arrested NAFLD-HCC growth. These results established METTL3 as an oncogenic factor in NAFLD-HCC.

NAFLD-HCC harbored a microenvironment that is largely different from virus-associated HCC. To define the NAFLD-HCC immune microenvironment upon METTL3 overexpression, we performed scRNA-seq of NAFLD-HCC from control and liver-specific METTL3 knockin mice. We demonstrated NAFLD-HCC intrinsic METTL3 impaired CD8$^+$ T cell antitumor response, with significant reductions in active CD8$^+$ T cell subsets (IFN-γ^+ and GZMB$^+$) in liver-specific METTL3 knockin mice. In contrast, METTL3 knockout potentiated antitumor immunity through upregulating cytotoxic CD8$^+$ T cells. In orthotopic NAFLD-HCC mouse models, antibody-mediated depletion of CD8$^+$ T cells abolished the effect of METTL3 knockout on tumor growth, implying that the pro-tumorigenic effect of METTL3 largely depends on its inhibitory effect on CD8$^+$ T cells. *In vitro* co-cultures of CD8$^+$ T cells with NAFLD-HCC cell conditioned medium revealed METTL3-dependent suppression of CD8$^+$ T cells. Taken together, our evidence suggests that tumor-intrinsic METTL3 restricts the activation and effector states of CD8$^+$ T cells to mediate immune escape and NAFLD-HCC progression. CD8$^+$ T cells are critical players in NAFLD-HCC. In line with our results, Leslie et al.[19] showed that anti-CD8α impaired CD8$^+$ T cell-mediated antitumor immunity in orthotopic NAFLD-HCC and compromised immunotherapeutic efficacy. On the other hand, depletion of CD8$^+$ T cells in the preventive setting protects against NASH-HCC by inhibiting aberrant activation of CD8$^+$PD-1$^+$ T cells and consequent inflammation, fibrosis, and tumorigenesis.[3] Hence, the role of CD8$^+$ T cells in NAFLD-HCC is context dependent.

We next explored molecular mechanisms underlying the effect of METTL3 in NAFLD-HCC and showed that METTL3-mediated immune suppression in NAFLD-HCC depends on the regulation of cholesterol metabolism. RNA-seq in NAFLD-HCC cell lines identified the cholesterol biosynthesis pathway as a top-depleted gene set after the knockout of METTL3. In cell lines and METTL3 knockin mice, overexpression of METTL3 induced accumulation of free cholesterol and cholesteryl esters, while METTL3 knockout exerted opposite effects. Integrative RNA-seq, m^{6}A-seq, and Ribo-seq further identified SCAP as the direct target of METTL3. SCAP positively regulates cholesterol biosynthesis by facilitating proteolytic activation and protein maturation of SREBP2,[20] a master transcription factor for cholesterol biosynthesis genes. By promoting SCAP m^{6}A modification and

(B) Naive T cells isolated from tumor-bearing mice spleen were co-cultured with conditioned medium from Hepa1-6 cells with or without METTL3 knockout for 72 h, and CD8$^+$ T cell function markers were determined by flow cytometry.
(C) Human CD8$^+$ T cells were co-cultured with conditioned medium from HKCI2 cells with or without METTL3 depletion for 72 h, and CD8$^+$ T cell function markers were determined by flow cytometry.
(D) Human CD8$^+$ T cells were co-cultured with conditioned medium from HKCI2 cells with or without METTL3 knockdown for 72 h. Cholesterol (0.2 μg/mL) or cholesteryl esters (0.1 μg/mL) were added as indicated. Quantification of IFN-γ^+ and GZMB$^+$ CD8$^+$ T cells from different groups by flow cytometry.
(E) Human CD8$^+$ T cells co-cultured with conditioned medium from HKCI10 cells with or without METTL3 overexpression for 72 h. β-Cyclodextrin (β-CD, 0.5 mM) was added as indicated. Quantification of IFN-γ^+ and GZMB$^+$ CD8$^+$ T cells from different groups by flow cytometry.
(F) T cell-mediated tumor-killing assay. T cells isolated from Hepa1-6 orthotopic NASH liver were co-cultured with adherent tumor cells (Hepa1-6) in the presence of indicated cholesterol for 48 h, and the remaining tumor cells were measured by MTT. MTT, 3-(4,5-dimethylthiazoly-2-yl)-2,5-diphenylte-trazolium bromide.
(G) The statistical graph of the percentage of tumor cell killing in (F). T cells in the presentation of high cholesterol killed significantly fewer tumor cells than control.
(H) Human CD8$^+$ T cells were co-cultured with conditioned medium from METTL3-overexpressing HKCI2 cells with or without siSCAP for 72 h, and CD8$^+$ T cell function markers were analyzed by flow cytometry.
(I) Human CD8$^+$ T cells were co-cultured with conditioned medium from METTL3-overexpressing HKCI10 cells with or without siSCAP for 72 h, and CD8$^+$ T cell function markers were analyzed by flow cytometry.
Data are presented as mean ± SD. Each spot represents one subject. Statistical significance was determined by one-way ANOVA or two-sided Student's t test where appropriate. Ctrl, control; Cho, cholesterol; E, cholesteryl esters. See also Figure S6.

CellPress
OPEN ACCESS

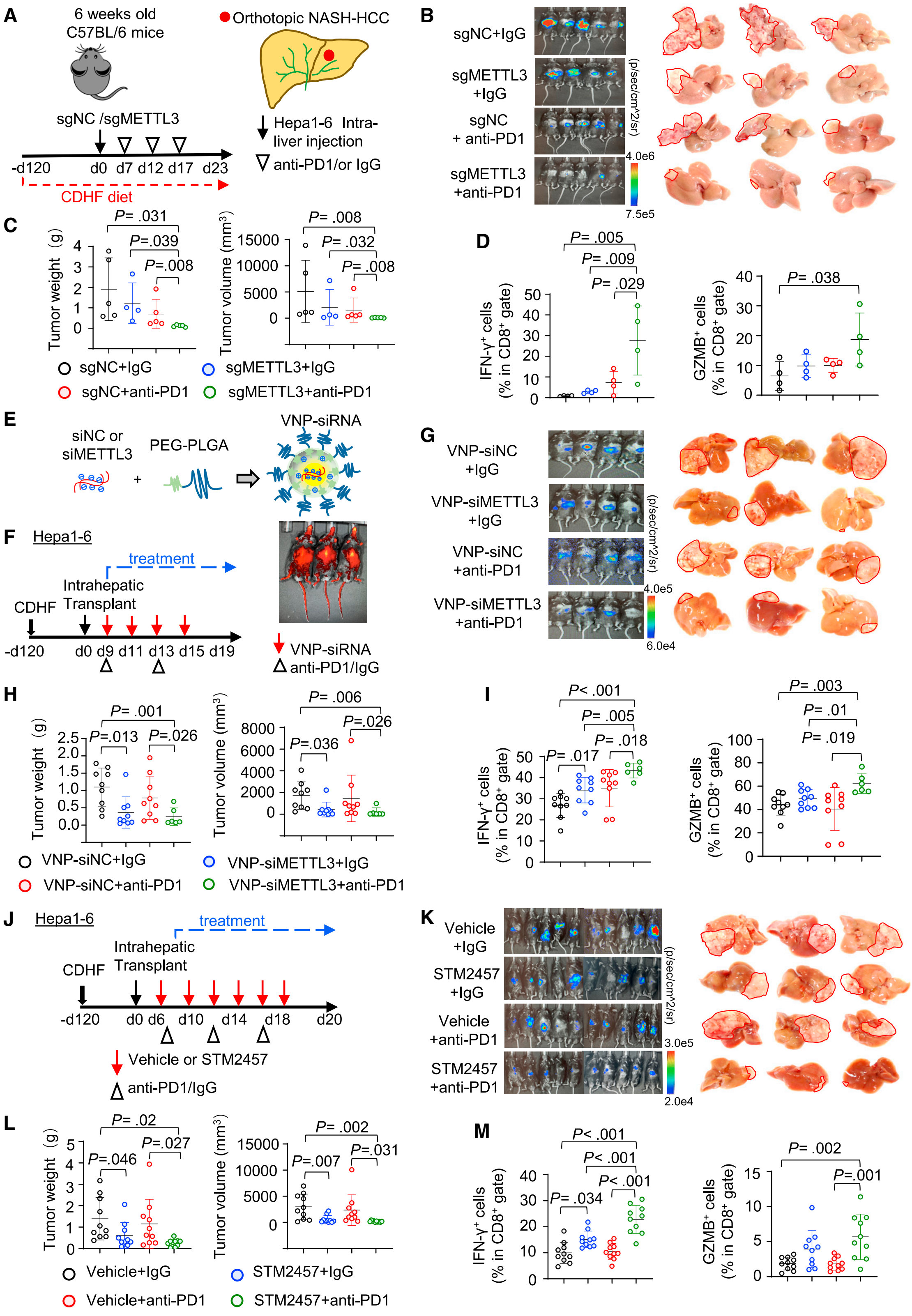

(legend on next page)

translation, METTL3 accelerates *de novo* cholesterol biosynthesis in NAFLD-HCC *in vitro* and in mice. Accordingly, SCAP depletion abolished cholesterol-inducing effect of METTL3. We thus identified a METTL3-m^{6}A-SCAP-cholesterol axis in NAFLD-HCC.

Cholesterol is a lipotoxic molecule and our previous work has established vital roles of cholesterol in NAFLD-HCC[11,13,21] by promoting cell proliferation, inflammation, and dysbiosis. We thus asked if cholesterol underlies the immunosuppressive effect of METTL3 in NAFLD-HCC. Indeed, we demonstrated that cholesterol depletion reversed the impairment of cytotoxic CD8$^+$ T cells by NAFLD-HCC cells overexpressing METTL3. In contrast, exogenous cholesterol or cholesteryl esters addition reversed METTL3 knockout-induced CD8$^+$ T cell activation. Corroborating these results, SCAP knockdown in METTL3 overexpressing NAFLD-HCC cells reinvigorated cytotoxic T cell response. In support of this, T cell-killing assays revealed that CD8$^+$ T cells extracted from tumors of mice with NAFLD-HCC exhibited an inhibitory effect on tumor cells. Furthermore, we found that cholesterol supplementation dose-dependently compromised the cancer cell-killing ability of tumor-infiltrating CD8$^+$ T cells. In agreement with our findings, others have shown that cholesterol accumulation in the tumor microenvironment could induce CD8$^+$ T cell exhaustion through endoplasmic reticulum (ER) stress.[15] In summary, we demonstrate that METTL3-mediated m^{6}A modification of SCAP rewires cholesterol metabolism to impair CD8$^+$ T cell antitumor response, thereby promoting NAFLD-HCC growth.

HCC patients typically have poor survival rates because of a lack of effective treatment. The advent of anti-PD-1 therapy has revolutionized HCC treatment,[22] with durable clinical response in 15%–30% of HCC patients. Nevertheless, a substantial portion of HCCs failed to respond to anti-PD-1 therapy. In particular, two landmark studies showed that anti-PD-1 treatment failed to reinvigorate CD8$^+$ T cell tumor surveillance but paradoxically activated Cxcr6$^+$ auto-aggressive CD8$^+$TNF$^+$PD-1$^+$ T cells, which induce liver damage, cirrhosis, and HCC.[3,4] Identification of additional strategies that potentiate anti-PD-1 therapy is urgent and important. Our work here demonstrated that METTL3 plus anti-PD-1 is a therapeutic combination that effectively inhibits the growth of NAFLD-HCC. We showed that anti-PD-1 treatment in METTL3 knockout NAFLD-HCC tumors provoked tumor regression by restoring IFN-γ^+ and GZMB$^+$ CD8$^+$ T cells. Moreover, the targeting of METTL3 using nanoparticle-siRNA or METTL3-specific inhibitor (STM2457) synergized with anti-PD-1 treatment to reactivate CD8$^+$ T cells and suppress the growth of orthoptic NAFLD-HCC tumors. Our study suggests METTL3 as a potential therapeutic target for combination therapy with ICB therapy for NAFLD-HCC treatment.

It has been reported that METTL3 is upregulated in virus-associated HCC,[23,24] and oncogenic pathways activated in HCC, such as MAPK/ERK, have been shown to promote METTL3 expression.[24] Although we identified the role of METTL3 in NAFLD-HCC, its function in NAFLD remains incompletely understood. Li et al. showed that hepatocyte-specific *Mettl3* deletion at an early age in mice leads to liver injury and NAFLD.[25] However, the complete ablation of *Mettl3* in neonatal mouse liver leads to nuclear pleomorphism and ballooning as well as lethality phenotype within 7 weeks.[26,27] This implies that METTL3 is crucial for normal liver development, and its ablation cannot accurately represent a physiologically relevant model for NAFLD and its progression to NASH and NASH-HCC. Our hepatocyte-specific *Mettl3* knockin model and the whole-body *Mettl3*$^{+/-}$ knockout model suggest that METTL3 promotes NAFLD-HCC, particularly at the tumor stage. Future studies using conditional, hepatocyte-specific *Mettl3* KO mouse model by crossing *Mettl3*$^{flox/flox}$ mice with *Alb-Cre*^{ERT2} will fully address the effect of liver-intrinsic *Mettl3* loss on NAFLD-HCC development.

In conclusion, we demonstrate that the m^{6}A writer METTL3 induces m^{6}A-SCAP-cholesterol axis to suppress the activation of antitumor CD8$^+$ T cells, thereby promoting NAFLD-HCC. The genetic or pharmacological blockade of METTL3 plus anti-PD-1 shows promising efficacy against NAFLD-HCC *in vivo*, emphasizing METTL3 as a potential therapeutic target for NAFLD-HCC treatment.

Limitations of the study

Although we illustrated the importance of METTL3 in NAFLD-HCC progression by using liver-specific *Mettl3* knockin mice

Figure 7. Targeting METTL3 reverses resistance to immunotherapy in NAFLD-HCC

(A) Experimental design for the orthotopic NAFLD-HCC model and anti-PD-1 treatment. C57BL/6J mice were inoculated with Hepa1-6-sgNC or Hepa1-6-sgMETTL3 cells and then treated with anti-PD-1 or IgG control.
(B) Bioluminescent imaging and representative images of mouse livers.
(C) Tumor weight and volume at endpoint (n = 4–5/group).
(D) Quantification of tumor-infiltrating IFN-γ^+ and GZMB$^+$ CD8$^+$ T cells.
(E) Illustration of *in vivo* siRNA using PEG-PLGA-based nanoparticles (vesicle-like nanoparticle [VNP]-siRNA). PEG-PLGA, polyethylene glycol-poly lactic acid-co-glycolic acid.
(F) Experimental design for VNP-siRNA and anti-PD-1 treatment in orthotopic NAFLD-HCC. C57BL/6J mice inoculated with Hepa1-6 cells were treated with VNP-siMETTL3, anti-PD-1, or their combination.
(G) Bioluminescent imaging and representative images of mouse livers.
(H) Tumor weight and volume at endpoint (n = 6–9/group).
(I) Quantification of tumor-infiltrating IFN-γ^+ and GZMB$^+$ CD8$^+$ T cells.
(J) Experimental design for STM2457 and anti-PD-1 treatment in orthotopic NAFLD-HCC. C57BL/6J mice inoculated with Hepa1-6 cells were treated with STM2457, anti-PD-1, or their combination.
(K) Bioluminescent imaging and representative images of livers.
(L) Tumor weight and volume at endpoint (n = 10/group).
(M) Quantification of tumor-infiltrating IFN-γ^+ and GZMB$^+$ CD8$^+$ T cells.
Data are presented as mean ± SD. Each spot represents one subject. Statistical significance was determined by one-way ANOVA or two-sided Student's t test where appropriate. See also Figure S7.

CellPress
OPEN ACCESS

and heterozygous *Mettl3* knockout mice, these results will require validation in a conditional, hepatocyte-specific *Mettl3* knockout mouse model.

STAR★METHODS

Detailed methods are provided in the online version of this paper and include the following:

- KEY RESOURCES TABLE
- RESOURCE AVAILABILITY
  - Lead contact
  - Materials availability
  - Data and code availability
- EXPERIMENTAL MODEL AND SUBJECT DETAILS
  - Patients and samples
  - Cell lines
  - Animal models
- METHOD DETAILS
  - Lentiviral transduction of tumor cells
  - siRNA transfection
  - m^6A dot blot assay on total RNA
  - Multiplex immunofluorescence staining
  - N6-methyladenosine sequencing
  - Ribosome-sequencing
  - RNA sequencing
  - Single-cell RNA-Sequencing
  - Library generation, sequencing, and data analysis
  - RNA Immunoprecipitation-qPCR (RIP-qPCR)
  - Ribosome nascent-chain complex-bound mRNA-qPCR (RNC-qPCR)
  - Cholesterol cholesterol/cholesteryl ester concentrations
  - IFN-γ measurement
  - RT-qPCR
  - T cell coculture assay
  - T cell-mediated tumor-killing assay
  - Luciferase reporter assay
  - LDLR knockdown in T cells
  - Immunohistochemistry
  - Western blot
  - Serum AFP measurement
  - Serum biochemical assay for cholesterol, ALT, AST and TG
  - Colony formation assay
  - Apoptosis and cell cycle analysis
- QUANTIFICATION AND STATISTICAL ANALYSIS

SUPPLEMENTAL INFORMATION

Supplemental information can be found online at https://doi.org/10.1016/j.xcrm.2023.101144.

ACKNOWLEDGMENTS

This study was supported by the National Natural Science Foundation of China (NSFC 81972576), RGC Theme-based Research Scheme (T12-703/19-R), RGC Research Impact Fund (R4017-18F), RGC Collaborative Research Fund (C4039-19GF), RGC-GRF (14110819 and 14111621), and the Vice-Chancellor's Discretionary Fund CUHK (4930775).

AUTHOR CONTRIBUTIONS

Y.P. performed experiments and drafted the manuscript. H.C., X.Z., and C.C.W. commented on the study and revised the manuscript. W.L., Y.D., and D.H. performed bioinformatic analyses. J.Z., W.W., and D.C. assisted with the experiments. J.W., Y.Z., and C.L. performed animal experiments. A.H.K.C. performed a histological evaluation. N.W. provided human NAFLD-HCC cell lines. K.M. provided human NAFLD-HCC samples. J.Y. supervised the study and revised the manuscript.

DECLARATION OF INTERESTS

The authors declare no competing interests.

Received: September 12, 2022
Revised: May 31, 2023
Accepted: July 13, 2023
Published: August 15, 2023

REFERENCES

1. Huang, D.Q., El-Serag, H.B., and Loomba, R. (2021). Global epidemiology of NAFLD-related HCC: trends, predictions, risk factors and prevention. Nat. Rev. Gastroenterol. Hepatol. *18*, 223–238. https://doi.org/10.1038/s41575-020-00381-6.
2. Sangro, B., Sarobe, P., Hervás-Stubbs, S., and Melero, I. (2021). Advances in immunotherapy for hepatocellular carcinoma. Nat. Rev. Gastroenterol. Hepatol. *18*, 525–543. https://doi.org/10.1038/s41575-021-00438-0.
3. Pfister, D., Núñez, N.G., Pinyol, R., Govaere, O., Pinter, M., Szydlowska, M., Gupta, R., Qiu, M., Deczkowska, A., Weiner, A., et al. (2021). NASH limits anti-tumour surveillance in immunotherapy-treated HCC. Nature *592*, 450–456. https://doi.org/10.1038/s41586-021-03362-0.
4. Dudek, M., Pfister, D., Donakonda, S., Filpe, P., Schneider, A., Laschinger, M., Hartmann, D., Hüser, N., Meiser, P., Bayerl, F., et al. (2021). Auto-aggressive CXCR6(+) CD8 T cells cause liver immune pathology in NASH. Nature *592*, 444–449. https://doi.org/10.1038/s41586-021-03233-8.
5. Zaccara, S., Ries, R.J., and Jaffrey, S.R. (2019). Reading, writing and erasing mRNA methylation. Nat. Rev. Mol. Cell Biol. *20*, 608–624. https://doi.org/10.1038/s41580-019-0168-5.
6. He, L., Li, H., Wu, A., Peng, Y., Shu, G., and Yin, G. (2019). Functions of N6-methyladenosine and its role in cancer. Mol. Cancer *18*, 176. https://doi.org/10.1186/s12943-019-1109-9.
7. Chen, H., Gao, S., Liu, W., Wong, C.C., Wu, J., Wu, J., Liu, D., Gou, H., Kang, W., Zhai, J., et al. (2021). RNA N(6)-Methyladenosine Methyltransferase METTL3 Facilitates Colorectal Cancer by Activating the m(6)A-GLUT1-mTORC1 Axis and Is a Therapeutic Target. Gastroenterology *160*, 1284–1300.e16. https://doi.org/10.1053/j.gastro.2020.11.013.
8. Minton, K. (2014). RNA decay: stabilizing stemness through m(6)A. Nat. Rev. Mol. Cell Biol. *15*, 76–77. https://doi.org/10.1038/nrm3745.
9. Wang, S., Gao, S., Zeng, Y., Zhu, L., Mo, Y., Wong, C.C., Bao, Y., Su, P., Zhai, J., Wang, L., et al. (2022). N6-Methyladenosine Reader YTHDF1 Promotes ARHGEF2 Translation and RhoA Signaling in Colorectal Cancer. Gastroenterology *162*, 1183–1196. https://doi.org/10.1053/j.gastro.2021.12.269.
10. Han, D., Liu, J., Chen, C., Dong, L., Liu, Y., Chang, R., Huang, X., Liu, Y., Wang, J., Dougherty, U., et al. (2019). Anti-tumour immunity controlled through mRNA m(6)A methylation and YTHDF1 in dendritic cells. Nature *566*, 270–274. https://doi.org/10.1038/s41586-019-0916-x.
11. Liu, D., Wong, C.C., Fu, L., Chen, H., Zhao, L., Li, C., Zhou, Y., Zhang, Y., Xu, W., Yang, Y., et al. (2018). Squalene epoxidase drives NAFLD-induced

hepatocellular carcinoma and is a pharmaceutical target. Sci. Transl. Med. *10*, eaap9840. https://doi.org/10.1126/scitranslmed.aap9840.

12. Gao, S., Soares, F., Wang, S., Wong, C.C., Chen, H., Yang, Z., Liu, W., Go, M.Y.Y., Ahmed, M., Zeng, Y., et al. (2021). CRISPR screens identify cholesterol biosynthesis as a therapeutic target on stemness and drug resistance of colon cancer. Oncogene *40*, 6601–6613. https://doi.org/10.1038/s41388-021-01882-7.
13. Zhang, X., Coker, O.O., Chu, E.S., Fu, K., Lau, H.C.H., Wang, Y.X., Chan, A.W.H., Wei, H., Yang, X., Sung, J.J.Y., and Yu, J. (2021). Dietary cholesterol drives fatty liver-associated liver cancer by modulating gut microbiota and metabolites. Gut *70*, 761–774. https://doi.org/10.1136/gutjnl-2019-319664.
14. Yang, T., Espenshade, P.J., Wright, M.E., Yabe, D., Gong, Y., Aebersold, R., Goldstein, J.L., and Brown, M.S. (2002). Crucial step in cholesterol homeostasis: sterols promote binding of SCAP to INSIG-1, a membrane protein that facilitates retention of SREBPs in ER. Cell *110*, 489–500. https://doi.org/10.1016/s0092-8674(02)00872-3.
15. Ma, X., Bi, E., Lu, Y., Su, P., Huang, C., Liu, L., Wang, Q., Yang, M., Kalady, M.F., Qian, J., et al. (2019). Cholesterol Induces CD8(+) T Cell Exhaustion in the Tumor Microenvironment. Cell Metab. *30*, 143–156.e5. https://doi.org/10.1016/j.cmet.2019.04.002.
16. Wang, S., Gao, S., Zeng, Y., Zhu, L., Mo, Y., Wong, C.C., Bao, Y., Su, P., Zhai, J., Wang, L., et al. (2022). N6-Methyladenosine Reader YTHDF1 Promotes ARHGEF2 Translation and RhoA Signaling in Colorectal Cancer. Gastroenterology *162*, 1183–1196. https://doi.org/10.1053/j.gastro.2021.12.269.
17. Zhang, Y., Zhang, Z., Li, S., Zhao, L., Li, D., Cao, Z., Xu, X., and Yang, X. (2021). A siRNA-Assisted Assembly Strategy to Simultaneously Suppress "Self" and Upregulate "Eat-Me" Signals for Nanoenabled Chemo-Immunotherapy. ACS Nano *15*, 16030–16042. https://doi.org/10.1021/acsnano.1c04458.
18. Yankova, E., Blackaby, W., Albertella, M., Rak, J., De Braekeleer, E., Tsagkogeorga, G., Pilka, E.S., Aspris, D., Leggate, D., Hendrick, A.G., et al. (2021). Small-molecule inhibition of METTL3 as a strategy against myeloid leukaemia. Nature *593*, 597–601. https://doi.org/10.1038/s41586-021-03536-w.
19. Leslie, J., Mackey, J.B.G., Jamieson, T., Ramon-Gil, E., Drake, T.M., Fercoq, F., Clark, W., Gilroy, K., Hedley, A., Nixon, C., et al. (2022). CXCR2 inhibition enables NASH-HCC immunotherapy. Gut *71*, 2093–2106. https://doi.org/10.1136/gutjnl-2021-326259.
20. Brown, M.S., Radhakrishnan, A., and Goldstein, J.L. (2018). Retrospective on Cholesterol Homeostasis: The Central Role of Scap. Annu. Rev. Biochem. *87*, 783–807. https://doi.org/10.1146/annurev-biochem-062917-011852.
21. Liang, J.Q., Teoh, N., Xu, L., Pok, S., Li, X., Chu, E.S.H., Chiu, J., Dong, L., Arfianti, E., Haigh, W.G., et al. (2018). Dietary cholesterol promotes steatohepatitis related hepatocellular carcinoma through dysregulated metabolism and calcium signaling. Nat. Commun. *9*, 4490. https://doi.org/10.1038/s41467-018-06931-6.
22. Finn, R.S., Qin, S., Ikeda, M., Galle, P.R., Ducreux, M., Kim, T.Y., Kudo, M., Breder, V., Merle, P., Kaseb, A.O., et al. (2020). Atezolizumab plus Bevacizumab in Unresectable Hepatocellular Carcinoma. N. Engl. J. Med. *382*, 1894–1905. https://doi.org/10.1056/NEJMoa1915745.
23. Chen, M., Wei, L., Law, C.T., Tsang, F.H.C., Shen, J., Cheng, C.L.H., Tsang, L.H., Ho, D.W.H., Chiu, D.K.C., Lee, J.M.F., et al. (2018). RNA N6-methyladenosine methyltransferase-like 3 promotes liver cancer progression through YTHDF2-dependent posttranscriptional silencing of SOCS2. Hepatology *67*, 2254–2270. https://doi.org/10.1002/hep.29683.
24. Chen, S.L., Liu, L.L., Wang, C.H., Lu, S.X., Yang, X., He, Y.F., Zhang, C.Z., and Yun, J.P. (2020). Loss of RDM1 enhances hepatocellular carcinoma progression via p53 and Ras/Raf/ERK pathways. Mol. Oncol. *14*, 373–386. https://doi.org/10.1002/1878-0261.12593.
25. Li, X., Yuan, B., Lu, M., Wang, Y., Ding, N., Liu, C., Gao, M., Yao, Z., Zhang, S., Zhao, Y., et al. (2021). The methyltransferase METTL3 negatively regulates nonalcoholic steatohepatitis (NASH) progression. Nat. Commun. *12*, 7213. https://doi.org/10.1038/s41467-021-27539-3.
26. Barajas, J.M., Lin, C.H., Sun, H.L., Alencastro, F., Zhu, A.C., Aljuhani, M., Navari, L., Yilmaz, S.A., Yu, L., Corps, K., et al. (2022). METTL3 Regulates Liver Homeostasis, Hepatocyte Ploidy, and Circadian Rhythm-Controlled Gene Expression in Mice. Am. J. Pathol. *192*, 56–71. https://doi.org/10.1016/j.ajpath.2021.09.005.
27. Xu, Y., Zhou, Z., Kang, X., Pan, L., Liu, C., Liang, X., Chu, J., Dong, S., Li, Y., Liu, Q., et al. (2022). Mettl3-mediated mRNA m(6)A modification controls postnatal liver development by modulating the transcription factor Hnf4a. Nat. Commun. *13*, 4555. https://doi.org/10.1038/s41467-022-32169-4.
28. Polyzos, S.A., Kountouras, J., and Mantzoros, C.S. (2019). Obesity and nonalcoholic fatty liver disease: From pathophysiology to therapeutics. Metabolism *92*, 82–97. https://doi.org/10.1016/j.metabol.2018.11.014.

STAR★METHODS

KEY RESOURCES TABLE

REAGENT or RESOURCE	SOURCE	IDENTIFIER
Antibodies		
Anti-mouse CD4 (PE-Cy7) (RM4-5)	Biolegend	Cat# 100528; RRID: AB_312729
Anti-mouse CD279(BV421) (29F.1A12)	Biolegend	Cat# 135221; RRID: AB_2562568
Anti-mouse CD8a(PE-Cy5) (53-6.7)	Biolegend	Cat# 100710; RRID: AB_312749
Anti-mouse TNF-α (FITC) (MP6-XT22)	Biolegend	Cat# 506304; RRID: AB_315425
Anti-mouse IFN-γ(BV711) (XMG1.2)	Biolegend	Cat# 505836; RRID: AB_2650928
Anti-mouse/human Granzyme B(PE-Cy7) (NGZB)	eBioscience™	Cat# 25-8898-82
Anti-mouse CD45(BV605) (30-F11)	Biolegend	Cat# 103155; RRID: AB_2650656
Anti- human IFN-γ(BV711) (4S.B3)	Biolegend	Cat# 502540; RRID: AB_2563506
Anti-mouse CD3(PE) (17A2)	Biolegend	Cat# 100206; RRID: AB_312663
Anti-human CD8(PE-Cy5) (SK1)	Biolegend	Cat# 344770
Anti-mouse NK-1.1(PE-Cy7) (PK136)	Biolegend	Cat# 108714; RRID: AB_389364
Anti-mouse TNF-α(FITC) (MP6-XT22)	Biolegend	Cat# 506303; RRID: AB_315424
Anti-mouse/human Ki-67 (FITC)(11F6)	Biolegend	Cat# 151211; RRID: AB_2814054
Anti-β-actin	Cell Signaling	Cat# 4970; RRID: AB_2223172
Anti-METTL3	Cell Signaling	Cat# D2I6O
Anti-SQLE	Abcam	Cat# ab76896; RRID: AB_10562779
Anti-HMGCR	Abcam	Cat# ab242315; RRID: AB_2928124
Anti-SCAP	Thermo Fisher	Cat# PA5115869; RRID: AB_2900503
Anti-SCAP	Bethy1	Cat# A303-554A
Anti-N6-methyladenosine	Abcam	Cat# ab208577; RRID: AB_2916290
Anti-Foxp3	Cell Signaling	Cat# 12653S
Anti-YTHDC1	Proteintech	Cat# 29441-1-AP; RRID: AB_2918307
Anti-SREBP2	R&D Systems	Cat# AF7119SP
Anti-Ki-67	Cell Signaling	Cat# 9129; RRID: AB_2687446
Anti-Phospho-p44/42 MAPK(Erk1/2)	Cell Signaling	Cat# 9101L
Anti-Phospho-Jak1(Ty1034/1035)	Cell Signaling	Cat# 74129S
Anti-Phospho-p90RSK (Ser380)	Cell Signaling	Cat# 9341S
Anti-LDLR monoclonal antibody	thermofisher	Cat# MA5-23916
Anti-mouse IgG, HRP-linked	Cell Signaling	Cat# 7076S
Anti-rabbit IgG, HRP-linked	Cell Signaling	Cat# 7074S
Rabbit on Rodent HRP-Polymer	Biocare	Cat# RMR622H
Anti-mouse PD1(RMP1-14)	Bio-X-Cell	Cat# BE0146; RRID: AB_10949053
Rat IgG2α isotype control (2A3)	Bio-X-Cell	Cat# BE0089; RRID: AB_1107769
Anti-mouse CD8α (2.43)	Bio-X-Cell	Cat# BE0061; RRID: AB_1125541
Biological samples		
Buffy coats of healthy donors	Prince of Wales Hospital, the Chinese University of Hong Kong	N/A
Tumor and normal adjacent tissues from NAFLD-HCC patients	Queen Mary Hospital of the University of Hong Kong	N/A
Chemicals, peptides, and recombinant proteins		
Cholesterol	Sigma	Cat# C4951
β-cyclodextrin	Sigma	Cat# C4555
Human IL-2	Biolegend	Cat# 589104
Mouse IL-2	Biolegend	Cat# 575402

(*Continued on next page*)

Continued		
REAGENT or RESOURCE	SOURCE	IDENTIFIER
DNase I, Grade II	Roche	Cat# 10104159001
Critical commercial assays		
Cholesterol/Cholesteryl Ester Quantification kit	Abcam	Cat# ab65359
Magna RIPTM RNA-Binding Protein Immunoprecipitation	Sigma	Cat# 17-700
Mouse alpha-Fetoprotein/AFP Quantikine ELISA Kit	R&D Systems	Cat# MAFP00
STM2457	MedChemExpress	Cat# HY-134836
EasySep Mouse T cell Isolation Kit	STEMCELL	Cat# 19851
EasySep^{TM} Human T Cell Isolation Kit	STEMCELL	Cat# 17951
Dynabeads Mouse T-Activator CD3/CD28 for T cells	ThermoFisher	Cat# 11456D
Dynabeads Human T-Activator CD3/CD28 for T cells	ThermoFisher	Cat# 11161D
Magna ChIP Protein A+G Magnetic Beads	Sigma-Aldrich	Cat# 16-663
N,N- diethylnitrosamine	Sigma-Aldrich	Cat# 55-18-5
Lipofectamine RNAiMAX reagent	Invitrogen	Cat# 13778150
Lipofectamine 2000 Transfection Reagent	ThermoFisher	Cat# 11668500
MTT	Thermo Scientific	Cat# M6494
PrimeScript RT reagent Kit	Takara	Cat# RR037A
Permeabilization Buffer 10X	Invitrogen	Cat# 00-8333-56
IC Fixation Buffer 125 mL	eBiosciences	Cat# 00-8222-49
RBC Lysis Buffer (10X)	Biolegend	Cat# 420301
Cell Staining Buffer	Biolegend	Cat# 420201
Dual-Luciferase® Reporter Assay System, Firefly, Renilla	Promega	Cat# E1910
Ionomycin	STEMCELL	Cat# 73724
Phorbol 12-myristate 13-acetate (PMA)	Sigma-Aldrich	Cat# P1585
Monesin sodium salt	Sigma-Aldrich	Cat# M5273
D-Luciferin, Monopotassium Salt	Thermo Fisher	Cat# 88293
Deposited data		
Human NAFLD-HCC RNA sequencing data	European Genome-Phenome Archive	EGAD00001004326
M^{6}A-sequencing and RNA sequencing data	NCBI Gene expression omnibus (GEO)	GSE233808
Experimental models: Cell lines		
HKCI2	Kindly provided by Dr.Nathalie Wong	N/A
HKCI10	Kindly provided by Dr.Nathalie Wong	N/A
RIL-175	Kindly gifted by Prof.Lars Zender	N/A
Hepa1-6	ATCC	Cat#CRL-1830
Experimental models: Organisms/strains		
C57BL/6J	Jackson Laboratory	Cat# 000664
C57BL/6J, *Rosa26-lsl-Mettl3Alb-Cre*	Shanghai Model Organisms	In this study
C57BL/6J, *Mettl3* knocked-out	Cyagen	In this study
Oligonucleotides		
Human SCAP for Merip-qPCR<br>Forward: TGGTGTATGTGCCCTCTGTG<br>Reverse: CCCCAAGTCCAGGTTCAGT	This paper	In this study
Human β-actin for RT-PCR<br>Forward: CACCATTGGCAATGAGCGGTTC<br>Reverse: AGGTCTTTGCGGATGTCCACGT	This paper	In this study
Mouse β-actin for RT-PCR<br>Forward: CATTGCTGACAGGATGCAGAAGG<br>Reverse: TGCTGGAAGGTGGACAGTGAGG	This paper	In this study

(Continued on next page)

Continued

REAGENT or RESOURCE	SOURCE	IDENTIFIER
Human METTL3 for RT-PCR<br>Forward: CTATCTCCTGGCACTCGCAAGA<br>Reverse: GCTTGAACCGTGCAACCACATC	This paper	In this study
Mouse Mettl3 for RT-PCR<br>Forward: CAGTGCTACAGGATGACGGCTT<br>Reverse: CCGTCCTAATGATGCGCTGCAG	This paper	In this study
Human SCAP siRNA	Thermo Fisher	Cat# 4427038
Mouse sgMETTL3<br>Forward: CACCggactatcactacggaaggt<br>Reverse: AAACaccttccgtagtgatagtcc	This paper	In this study
Recombinant DNA		
Plasmid lentiCRISPRv2 vector	ADDgene	N/A
Software and algorithms		
GraphPad Prism 8.3.0	GraphPad Software	N/A
FlowJo 10.0	BD Biosciences	N/A
ImageJ	NIH	https://imagej.nih.gov/ij/
ImageLab	Biorad	N/A

RESOURCE AVAILABILITY

Lead contact

Further information and inquiries regarding resources and reagents should be directed to the lead contact, Dr. Jun Yu (junyu@cuhk.edu.hk), who will fulfill these requests.

Materials availability

This study did not generate new unique reagents.

Data and code availability

The sequencing data reported in this paper are deposited in NCBI Gene Expression Omnibus (GEO) database with accession number GEO: GSE233808 and will be publicly available as of the date of publication. The software and algorithms for data analyses used in this study are published and referenced throughout the STAR Methods section. No original code is featured in this paper. Any additional information required to reanalyze the data reported in this paper is available from the lead contact upon request.

EXPERIMENTAL MODEL AND SUBJECT DETAILS

Patients and samples

Human NAFLD-HCC tumor tissues and adjacent normal tissues were collected in Prince of Wales Hospital, the Chinese University of Hong Kong, from biopsy-proven NAFLD-HCC (n = 17). In the independent validation cohort, 10 paired NAFLD-HCC samples were collected at Queen Mary Hospital of the University of Hong Kong. Written informed consent was obtained from all subjects, and the study protocol was approved by the Clinical Research Ethics Committee of the Chinese University of Hong Kong and the University of Hong Kong.

Cell lines

Human NAFLD-HCC cell lines HKCI2 and HKCI10 were established from human NAFLD-HCC patients by Dr. Nathalie Wong from the Chinese University of Hong Kong.[11] Murine HCC cell line Hepa1-6 was purchased from the American Type Culture Collection (ATCC; CRL-1830). RIL-175 mouse HCC cell line was kindly provided by Prof. Lars Zender from University Hospital Tubingen, Germany.

Animal models

Transgenic mouse model

Conditional Mettl3 knockin mice (*Rosa26-lsl-Mettl3*) were established by the Shanghai Model Organisms (China). To achieve hepatocyte-specific knockin of METTL3, these mice were bred to Albumin Cre mice (Alb-Cre) to obtain *Rosa26-lsl-Mettl3Alb-Cre* (*Mettl3*^{LKI}) mice. Male *Mettl3*^{LKI} mice and wildtype littermates were injected with a single dose of N, N-diethylnitrosamine (DEN) at 14 days of age (5mg/kg). At 6 weeks, the mice were fed with choline-deficient and high-fat diet (CDHFD) (36.2% fat, 27.3% carbohydrate, 27.2% protein, without choline) (Research diet, New Brunswick, NJ) or high-fat high cholesterol diet (HFHCD) (43.7% fat,

36.6% carbohydrate, 19.7% protein, and 0.203% cholesterol) (Specialty Feeds, Glen Forrest, WA) for 21 weeks to establish NAFLD-HCC. Serum alpha-fetoprotein (AFP) and liver magnetic resonance imaging (MRI) were performed to monitor tumor formation and growth. All experimental procedures were approved by the Animal Ethics Committee of Chinese University of Hong Kong.

METTL3 knockout mouse model

Heterozygous whole-body *Mettl3* knockout mice (*Mettl3*$^{+/-}$) were generated by Cyagen (Taicang, China).[7] Mice at 14 days old were injected intraperitoneally with DEN (25mg/kg). Four weeks later, mice were treated with an HFHC diet or normal chow for 17 weeks. To achieve spontaneous NAFLD-HCC, *Mettl3*$^{+/-}$ and wildtype mice were fed with an HFHC diet for 45 weeks. Serum alpha-fetoprotein (AFP) and liver MRI were performed to monitor the tumor formation and growth. All experimental procedures were approved by the Animal Ethics Committee of Chinese University of Hong Kong.

Syngeneic mouse tumor models

METTL3 knockout or control Hepa1-6 and RIL-175 cells were incubated into the NASH livers of C57BL/6 mice fed CDHFD for 16 weeks. Anti-PD1(BE0146, Bio-X-Cell) or IgG isotype control (BE0089, Bio-X-Cell) were given every 5 days via intraperitoneal injection (100μg/mice, every 5 days). STM2457 (50mg/mice, every other day, MedChemExpress) was given via intraperitoneal injection. All experimental procedures were approved by the Animal Ethics Committee of Chinese University of Hong Kong.

CD8$^+$ T cell depletion experiments

For CD8$^+$ cell depletion, an orthotopic NAFLD-HCC model was constructed using Hepa1-6-sgnc-lucifrase cells or Hepa1-6-sgMETTL3-luciferase cells. Anti-CD8α antibody (clone 2.43; BioXCell) was intraperitoneal injected on Day -1,2,5,8,11 (200ug in 100 μL PBS per mouse for the first time, and 100μg/mice for the following doses at the indicated time). For the control group, the same amount of isotype-matched rat IgG2α antibody were injected at the same time.

A mouse model for VNP-siMETTL3 and anti-PD1 therapy

To improve the stability and *in vivo* visibility, all pyrimidine bases(C/U) in siNC and siMETTL3 were modified by 2'-O-methyl, carrying Cyanine 5 fluorescence. A siRNA-assisted assembly strategy for delivery of siRNA targeting METTL3 through PEG-PLGA based nanoparticles was used, named vesicle-like nanoparticle (VNP), while siNC served as control. C57BL6 wildtype mice will be given CDHF diet for 16 weeks prior to implantation. NAFLD-HCC mouse model was set up using Hepa1-6-luciferase cells after tumor cells' successful incubation was confirmed by luciferase imaging, mice were treated with siNC/siMETTL3 (2OD/mice, every other day) by tail vein injection, with or without anti-PD1 (100ug/mice, every 5 days) by intraperitoneal injection starting at day 9, isotype-matched rat IgG2α antibody set up as control. The siRNA sequence was as follows: siMETTL3: sense: 5'-CCAAGGAAGAGUGCAUGAATT-3', antisense:5'-UUCAUGC ACUCUUCCUUGGTT-3'; siNC: sense: 5'-UUCUCCGAACGUGUCACGUTT-3′, antisense: 5′-ACGUGACA CGUUCGGAGAATT-3'.

METHOD DETAILS

Lentiviral transduction of tumor cells

SgRNAs were designed by Massachusetts Institute of Technology CRISPR Design tool (http://crispr.mit.edu) and cloned into lenti-CRISPR v2 (Addgene). The full-length open reading frame of mouse METTL3 (NM_019721) was cloned into pCDH-CMV-MCS-EF1-Puro lentivector. Two shRNAs targeting METTL3 and a nontargeting RNA sequence serving as negative control were derived from Sigma-Aldrich. The sequences of the shRNAs were as follows: Mettl3-sh1: 5'- CCGGGCCAAGGAACAATCCATTGTTCTC GAGAA CAATGGATTGTTCCTTGGCTTTT-3'; Mettl3-sh2:5'-CCGGGCAAGTATGTT CACTATGAAACTCGAGTTTCATAGTGACATACTTGCT TTTTG-3'.

For lentivirus production, 8 μg of plasmid DNA, 6 μg of psPAX2 (Addgene #12260), 2 μg of pMD2.G (Addgene #12259), and 36 μL of lipofectamine 2000 (Life Tech) were mixed for 20 min and subsequently added to 293T cells in 10 cm culture dish with 10ml DMEM. After 8 h incubation, the medium was replaced by fresh 15 ml DMEM for 48 h incubation. The supernatant was harvested to a 50ml centrifuge tube. To get the cleared supernatant, 0.45um filter was used. Finally, the virus aliquot and storage at -80°C.

siRNA transfection

SCAP siRNA (siSCAP) and negative control (siNC) were obtained from Thermo Scientific. Lipofectamine RNAiMAX Reagent (Invitrogen) was used for transfection.

m^{6}A dot blot assay on total RNA

Equal amounts of RNA were spotted to a nylon membrane (Thermo Fisher Scientific), followed by UV crosslinking for 30 minutes. Anti-N6-methyladenosine (m^{6}A) antibody (Abcam, ab208577) was used in the assay.

Multiplex immunofluorescence staining

Dissected tumor tissue was incubated in the PBS supplied with 1% bovine serum albumin (BSA), 1% collagenase D (Sigma) and 0.005% Dnase I (Sigma), then incubated at 37°C with shaking at 225rpm for 30 min. The digested tumor tissue was gently ground with a 10 mL syringe plunger on the 70 μm Cell-Strainer (BD Biosciences, San Jose, CA). The suspension was centrifuged at 700g, 4°C for 10 min. Then the pallets were collected and resuspended using 1% BSA for blocking. After 30 min incubation, cells were stained with fluorescence antibodies for 30 min at 4°C.

For CD8+ T cells and intracellular staining (IFN-γ and Granzyme B), cells were incubated with RPMI-1640 medium supplied with PMA (50 ng/mL; Sigma), ionomycin (1 mg/mL; Sigma), monensin (10 ng/mL; Sigma) for 4 h at 37°C in a 5% CO2 incubator, followed by staining for extracellular markers. After fixation and permeabilization via Intracellular Fixation & Permeabilization Buffer Set (Thermo Fisher Scientific, Waltham, MA), the cells were stained with intracellular molecules antibody in permeabilization buffer overnight. The fluorescent antibodies used in this study are shown in key resources table. After fluorescent antibody incubation, cells were washed with PBS and resuspended in 500 μL PBS buffer. Finally, cells were analyzed on BD FACSCelestaTM flow cytometer (BD Biosciences, San Jose, CA), and the data was run with FlowJo software (Treestar, Inc., San Carlos, CA).

N6-methyladenosine sequencing

m^6A-seq was performed as described in a previous publication.[7] RNA was extracted using Trizol reagent (Thermo Fisher Scientific) and treated with DNase I (Roche Diagnostics) to remove DNA contamination. RNA was subjected to fragmentation and then incubated with an anti-m^6A antibody (Merck Millipore). For m^6A-sequencing, the m^6A-enriched RNA was eluted from the beads, purified using RNeasy Mini Kit (Qiagen), and then sent for sequencing with the Illumina NovaSeq 6000 platform. For methylated RNA immunoprecipitation (MeRIP)–quantitative polymerase chain reaction (qPCR), m^6A-enriched RNA was reverse-transcribed by using the High-Capacity cDNA Reverse Transcription Kit (Thermo Fisher Scientific). The enrichment of m^6A-containing transcripts was determined by qPCR. The primers used for merip-qPCR in key resources table.

The m^6A Viewer software was used to analyze the m^6A peaks from sequencing data. The 5′ untranslated region (UTR), coding sequence, and 3′ UTR of each transcript were normalized to the same length, and the global profile of m6A peaks was evaluated. The m^6A motif was identified through DREME (http://meme-suite.org/tools/dreme) analysis. The differential m^6A level was analyzed accordingly.Integrative Genomics Viewer, version 2.3.94, was used for visualizing selected genes.

Ribosome-sequencing

Cycloheximide (Sigma, St Louis, MO, USA) was added to cell cultures medium to a final concentration of 100 μg/ml and incubated for 2 min. The resuspended extract in the lysis buffer was transferred to a new microtube and incubated on ice for 10 min. Cells were then triturated ten times through a 26-G needle. The lysate was centrifuged at 4°C and 20,000 g for 10 min, and the supernatant was collected. To prepare ribosome footprints (RFs), 10μL of RNase I (NEB, Ipswich, MA, USA) and 6μL of DNase I (NEB, Ipswich, MA, USA) were added to 400μL of lysate, which was then incubated for 45 min at room temperature with gentle mixing on a Nutator mixer. Nuclease digestion was stopped by adding 10μL of SUPERase·In RNase inhibitor (Ambion, Austin, TX, USA). Size exclusion columns (illustra MicroSpin S-400 HR Columns; GE Healthcare; catalog no. 27- 5140-01) were equilibrated with 3 mL of polysome buffer by gravity flow and centrifuged at 600g for 4 min at room temperature. 100μL of digested RFs were added to the column and centrifuged at 600g for 2 min. Next, 10μL 10% (wt/vol) SDS was added to the elution, and RFs with a size greater than 17nt were isolated according to the RNA Clean and Concentrator-25 kit (Zymo Research; R1017). To remove rRNA, short (50–80 bases) antisense DNA probes complementary to rRNA sequences were added to the solution containing RFs, then RNase H (NEB, Ipswich, MA, USA) and DNase I (NEB, Ipswich, MA, USA) were added to digest rRNA and residual DNA probes. Finally, RFs were further purified using magnet beads (Vazyme, Nanjing, Jiangsu, China). After obtaining the ribosome footprints above, Ribo-seq libraries were constructed using NEBNext® Multiple Small RNA Library Prep Set for Illumina® (catalog no. E7300S, E7300L). Briefly, adapters were added to both ends of RFs, followed by reverse transcription and PCR amplification. The 140-160bp size PCR products were enriched to generate a cDNA library and sequenced using Illumina HiSeqTM X10 by Gene Denovo Biotechnology Co. (Guangzhou, China)

RNA sequencing

RNA was extracted and treated with DNase I. After passing quality control, the libraries were generated and sequenced with the Illumina HiSeq 4000 (PE150) according to the protocol from the company (Epibiotek). RNA-seq reads were preceded by removing adapters using cutadapt, version 1.18, and mapped on the human reference (GENCODE, version 30) by HISAT2, version 2.1.0, with the default options. The number of reads mapped to each of genes was counted by using HTSeq, version 0.11.2, with the option -m intersection-nonempty. Gene expression levels were calculated as fragments per kilobase of transcript per million mapped reads using DESeq2.

Single-cell RNA-Sequencing

CD45+ tumor-infiltrating leukocytes from *Mettl3*^{LKI} or WT mice were enriched for single-cell analysis. Prior to magnetic enrichment, tumors were cut into small pieces of 1-4 mm^3, and put in PBS containing 1% BSA, 1% collagenase D (Sigma) and 0.005% Dnase I (Sigma), then incubated at 37°C with shaking at 225rpm for 15 min. After washing in a 50ml centrifuge tube by adding 10 ml PBS, and centrifuging at 400× g for 10 min, the cell pellet was resuspended in 40% Percoll (GE Healthcare). The cell suspension was gently layered over 70% Percoll gradient and centrifuged for 30 min at 1260× g. Leukocytes were obtained from the interface layer. Cells were diluted with Trypan Blue and counted using a hemocytometer. Tumors from five different mice were pooled per sample, and two samples were prepared for *Mettl3*^{LKI} or WT mice, respectively. For a target recovery of 5,000 single cells, 8-9,000 live cells were loaded onto the Chromium Controller (10X genomics) and processed according to the manufacturer's instructions. 2 samples were sequenced on an Illumina NextSeq500 sequencer using a 75-bp kit with paired-end reads.

Library generation, sequencing, and data analysis

Library construction was performed using standard 10x Genomics single-cell V(D)J+5' expression library construction procedures. Briefly, Gel beads, cell suspension, and enzyme reagents were added to the channel of Chromium Next GEM Chip G chip. Single cells and Gel beads were wrapped by oil beads to form gel beads (GEMs), which became a separate reaction system. After the formation of GEMs, oligonucleotides containing Illumina R1 sequencing primer, 16 nt 10x Barcode, 10 nt unique sub-identifier (UMI) and 13nt template switching oligonucleotide (TSO) on Gel beads were released and combined with cell lysates Mix with Master Mix. After incubation, mRNAs with poly(A) form full-length carrying 10x Barcode. After the reverse transcriptome was completed, the GEM-reverse transcriptome reaction mixture was recovered, and all GEMs are broken and mixed. The full-length cDNA is then PCR amplified to obtain sufficient product for T cell, B cell enrichment library or 5' gene expression library construction. Finally, Illumina sequencing library construction was performed.

The quality of raw sequencing data was checked by using FastQC. Based on high-quality sequencing data, Cellranger was used to perform sequencing data quality statistics, and to calculate the number of cells captured by the experiment and the number of genes detected. The single-cell transcriptome data was then analyzed using the Seurat pipeline. Specifically, we filtered out cells with a proportion of mitochondrial genes higher than 10%, normalized the raw count matrix, and selected the top 2000 genes with the most variation. For each cell, we divided the gene counts by the total counts and then multiplied by 10,000, followed by natural log transformation. FindVariableFeatures was used to identify hypervariable genes. To eliminate batch effects, following Seurat's recommended workflow for batch effect correction, we scaled each dataset, selected 2000 hypervariable genes as input to compute integration anchors (FindIntegrationAnchors), and then integrated (IntegrateData) batches. Multiple datasets are then integrated by searching for "anchors" between them, allowing us to explore shared cell types presented across different datasets and conditions. The integrated data were scaled and then subjected to principal component analysis (PCA), and the top 20 principal components (PCs) were retained for further analysis. The selected PCs were also used to compute the nearest neighbor graph and to cluster the cells with the t-distributed stochastic neighbor embedding (t-SNE) method. The cell types are annotated by the expression of known marker genes.[3,28]

RNA Immunoprecipitation-qPCR (RIP-qPCR)

RIP-qPCR was performed by Magna RIP^{TM} RNA-Binding Protein Immunoprecipitation (Sigma). In brief, cell pellets were lysis by RIP Lysis Buffer and stocked at -80°C. ~5 μg of the antibody of interest was incubated with 50 μL of magnetic beads suspension for 30 min at room temperature with rotation, IgG as control. Thaw the RIP lysate and centrifuge at 14,000 rpm for 10 minutes at 4°C. Removed 100 μL of the supernatant and added to each beads-antibody complex in RIP Immunoprecipitation Buffer for the immunoprecipitation of RNA-binding protein-RNA complexes. qPCR was performed with primers of SCAP listed in key resources table.

Ribosome nascent-chain complex-bound mRNA-qPCR (RNC-qPCR)

Cells were pre-treated with cycloheximide at the concentration of 100ug/ml for 15 min at 37°C, and then lysed in lysis buffer on ice for 30 min. Lysis buffer was prepared by adding 1% Triton X-100 into ribosome buffer, including 20mM HEPES-KOH (pH=7.4), 15mM $MgCL_2$, 200mM KCl, 100mg/ml cycloheximide and 2mM dithiothreitol). Cell lysate was extracted by centrifuging at 4°C for 5 hours at 190,000g. The cell pellets were harvested and extracted by TRIzol. Total RNA isolated from the input control and RNC samples were used for RT-qPCR for further analysis.

Cholesterol cholesterol/cholesteryl ester concentrations

Cells (10^6) or tissues (2 mg) were collected. Cholesterol/cholesteryl ester concentrations were detected by Cholesterol/Cholesteryl Ester Quantification kit (ab65359, Abcam).

IFN-γ measurement

Serum from mice was collected for 23 mouse cytokines using Bio-Plex Pro Mouse Cytokine 23-Plex immunoassay (Bio-Rad).

RT-qPCR

DNA was synthesized from total RNA using Transcriptor Reverse Transcriptase (Takara Bio., Mountain View, CA). Primers used in this study are shown in key resources table.

T cell coculture assay

For T cell coculture assay, T cells were isolated by the EasySepTM Mouse T cell Isolation Kit (STEMCELL). Isolated T cells were cultured with conditional medium collecting from Hepa1-6 cells, supplied with mouse T-activator CD3/CD28 Dynabeads (Invitrogen) and mouse IL-2 (Biolegend, 10ng/ml) for 72 hours. Human peripheral blood $CD8^+$ T cells were obtained from STEMCELL (#70027). Human $CD8^+$ T cells were co-cultured with conditional medium collecting from different NASH-HCC cells, with human T-activation CD3/CD28 Dynabeads (Invitrogen) and human IL-2 (R&D Systems, 10ng/ml) for 72 hours, with or without cholesterol (Sigma),

cholesterol ester (Sigma) or β- cyclodextrin (Sigma). Quantification of IFN-γ $^{+}$ and $GZMB^{+}$ $CD8^{+}$ T cells (among $CD8^{+}$ T cells) from different groups was conducted. T cells were stimulated with PMA, monensin, and ionomycin for 4 hours. IFN-γ or GZMB-producing cells were determined by flow cytometry.

T cell-mediated tumor-killing assay

To measure the cytotoxicity of T cells, T cells were isolated from orthotopic NAFLD-HCC liver using Hepa1-6 in NASH liver as described above, then activated and expanded with mouse T-activator CD3/CD28 Dynabeads (Invitrogen) and mouse IL-2 (Biolegend, 10ng/ml) for 72 hours. Hepa1-6 tumor cells were seeded in 96-well plates 1 day before the coculture treatment (1000 cells/well), then T cells were mixed at the ratio of 1:1 in the killing medium (RPMI 1640, 2% FBS), treated with cholesterol (Sigma) at different concentration (0, 0.25ug/ml, 0.75ug/ml,1 ug/ml). After 48h of coculture, the wells were washed twice with PBS to remove the T cells. Cell viability of the remaining tumor cells was measured by 3-(4,5-dimethylthiazoly-2-yl)-2,5-diphenylte-trazolium bromide (MTT) assay.

Luciferase reporter assay

To check the interaction between SCAP and METTL3, luciferase reporter assay was performed with the Dual-Luciferase Reporter Assay System (Promega) according to the manufacturer's description. SCAP 3'-UTR was cloned into pmiGLO luciferase reporter plasmid (Promega). Empty vector or SCAP 3'-UTR were transfected into cells using FuGENE ®HD (Promega). pRLTK Renilla Luciferase vector was co-transfected as an internal control. The relative luciferase activities were accessed 24 h post-transfection by Dual-Luciferase Reporter Assay System (Promega).

LDLR knockdown in T cells

To knockdown LDLR in T cells, T cells were spin-infected with the siLDLR (key resources table) and Lipofectamine RNAiMAX Reagent (Invitrogen) for 2h at 2,000 rpm with 10ng/ml IL-2. Spin-infection was repeated on day 2. The knockdown efficiency of LDLR expression on the cell surface was measured by flow cytometry.

Immunohistochemistry

Paraffin-embedded tissue slides were deparaffinized by xylene, followed by 100%, 90%, 70% ethanol, and finally, water. Dewaxed slides were immersed in preheated sodium citrate buffer in the microwave for 30 min for antigen retrieval, then cooled down at room temperature and washed with PBS for 2 min. To reduce non-specific background staining caused by endogenous peroxidase, slides were incubated in 3% hydrogen for 15 minutes with shaking, then washed using PBS for 5 min. Blocking reagent in IHC Select HRP/DAB kit (Merck, Germany) for 1 hour to block non-specific background staining. Blocking solution was removed, and the primary antibody working solution, Ki-67 (Cell Signaling Technology, #9027), was added to the slides at 4°C overnight. Then three times' wash with TBST were performed, and the slides were incubated with Rabbit-on-Rodent HRP-Polymer (Biocare Medical) at room temperature for 30 min. After washing with TBST three times, slides were incubated with DAB (Merck, Germany) for 5 min.

Western blot

Total protein was extracted using the CytoBuster protein extraction kit (Novagen, Austin, TX) containing protease inhibitors (Roche) and phosphatase inhibitors (Roche). The antibodies used in this study were listed in the key resources table.

Serum AFP measurement

Mouse serum AFP was detected by a mouse AFP/AFP ELISA kit (MAFO00, R&D Systems).

Serum biochemical assay for cholesterol, ALT, AST and TG

The ALT, AST, cholesterol and TG concentrations were detected by the Catalyst One Chemistry Analyzer according to the manufacturer's instructions (IDEXX Laboratories, Westbrook, Maine). 30 μL of serum from mice was diluted to 90 μL by physiological saline buffer. The diluted samples were automatically analyzed by the machine with ALT, AST, cholesterol and TG analysis slides, respectively.

Colony formation assay

For colony formation assays, stably transfected cells (1000 per well for HKCI10 and 800 per well for HKCI2) were seeded in six-well plates. After 7-9 days of culture, cells were fixed with 70% ethanol and stained with 0.5% crystal violet solution.

Apoptosis and cell cycle analysis

Apoptosis was assessed using the annexin-phycoerythrin/7-aminoactinomycin D staining kit (BD Biosciences). For cell cycle analysis, cells were serum-starved overnight and stimulated with complete medium for 4 to 8 hours. Cells were fixed in 70% ethanol, stained with propidium iodide, and analyzed by flow cytometry.

QUANTIFICATION AND STATISTICAL ANALYSIS

Statistics were performed using SPSS or GraphPad Prism 8.3 software. The results were represented as mean ± standard deviation (SD). Mann-Whitney U test or unpaired Student's *t*-test was performed to compare the variables of the two sample groups where appropriate. Kruskal-Wallis test and One-way analysis of variance (ANOVA) were used to compare the differences among multiple groups. The Pearson correlation coefficient was used to evaluate the correlation between two genes' expressions. The difference in cell viability was determined by repeated-measures ANOVA. Differences with *P* value are denoted in figures.

Cell Reports Medicine, Volume *4*

Supplemental information

METTL3 drives NAFLD-related hepatocellular carcinoma and is a therapeutic target for boosting immunotherapy

Yasi Pan, Huarong Chen, Xiang Zhang, Weixin Liu, Yanqiang Ding, Dan Huang, Jianning Zhai, Wenchao Wei, Jun Wen, Danyu Chen, Yunfei Zhou, Cong Liang, Nathalie Wong, Kwan Man, Alvin Ho-Kwan Cheung, Chi Chun Wong, and Jun Yu

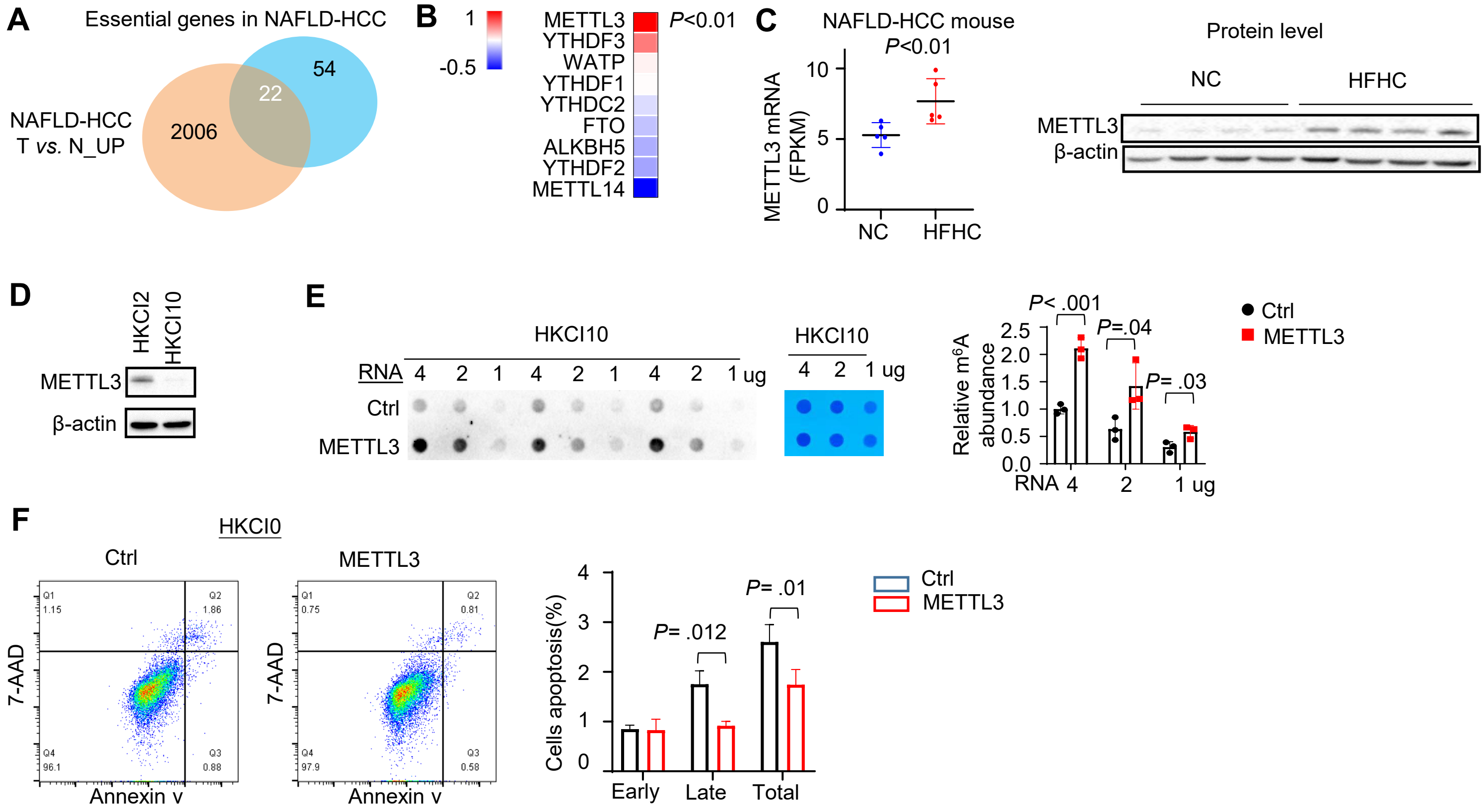

Figure S1. METTL3 is up-regulated in NAFLD-HCC patients and NAFLD-HCC mouse model. Related to Figure 1. (**A**) Integrated analysis of genes 1) increased in NAFLD-HCC in RNA-seq of 17 paired NAFLD-HCC and non-tumor tissues and 2) essential for NAFLD-HCC growth; (**B**) METTL3 is the top up-regulated m^{6}A regulator in 17 paired NAFLD-HCC patients; (**C**) METTL3 mRNA was up-regulated in dietary (HFHC) treated NAFLD-HCC animal model by RNA-seq, METTL3 protein was also upregulated. (**D**) Basal protein levels of METTL3 in two NAFLD-HCC cell lines. (**E**) Dot blot quantification of m^{6}A abundance in mRNA transcripts in HKCI10 cells overexpressing METTL3 (*left*). MB was used as a loading control (*middle*). Relative m^{6}A abundance in mRNA transcripts in HKCI10 cells (*right*). (**F**) METTL3 overexpression decreased apoptosis in HKCI10 cells as determined by annexin V–phycoerythrin and 7-AAD staining and flow cytometry. Data are presented as mean ± SD. Statistical significance was determined by one-way ANOVA or two-sided Student's *t*-test where appropriate.

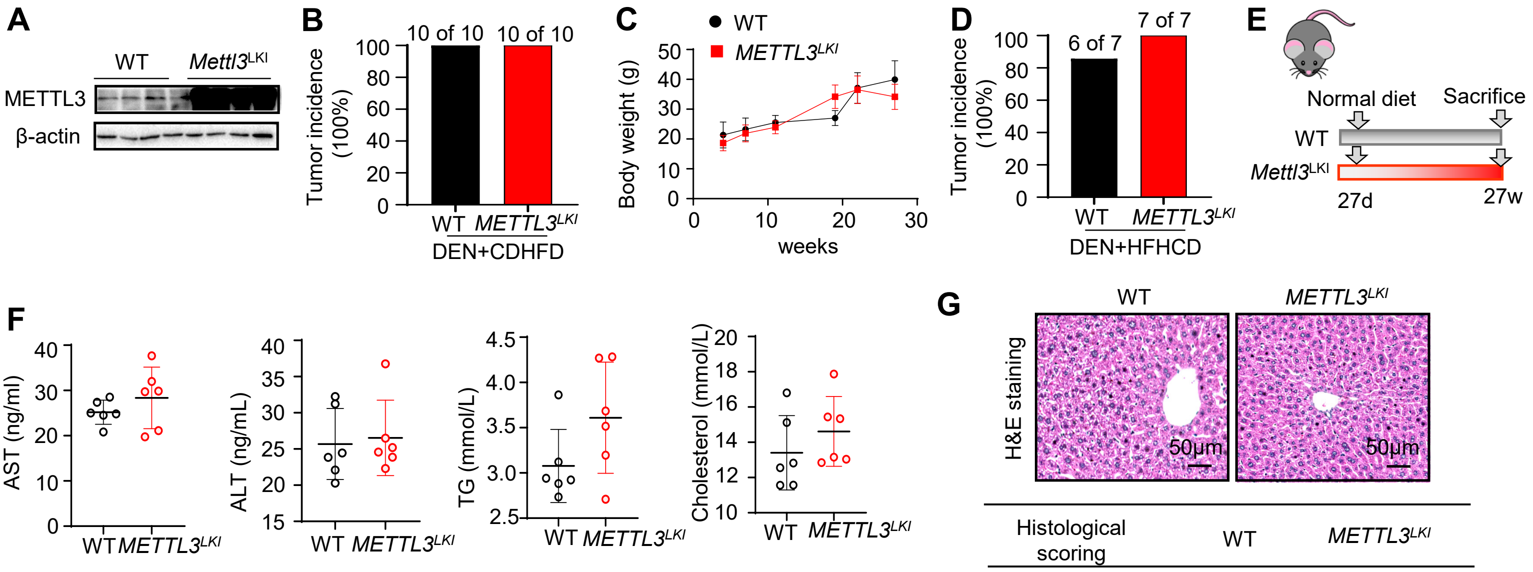

Histological scoring	WT	METTL3^LKI
Steatosis	0	0
Inflammation	0.02±0.12	0.02±0.08

Figure S2. Hepatocyte-specific METTL3 knockin accelerates diet-induced NAFLD-HCC. Related to Figure 2. (**A**) Western blot confirmed overexpression of METTL3 in the livers of *Mettl3*^{LKI} mice. (**B**) NAFLD-HCC tumor incidence in WT and *METTL3*^{LKI} mice treated with DEN plus CDHFD. (**C**) Body weight development in WT and *METTL3*^{LKI} mice treated with DEN plus CDHFD. (**D**) NAFLD-HCC tumor incidence in WT and *METTL3*^{LKI} mice treated with DEN plus HFHC diet. (**E**) Experimental schematic of WT and *METTL3*^{LKI} mice were fed with normal diet for 27 weeks (n=6/group) . (**F**) Serum AST, ALT, TG and cholesterol level in WT and *METTL3*^{LKI} mice treated with normal diet at 27 weeks. (**G**) Representative images of H&E staining in WT and *METTL3*^{LKI} mice fed normal diet at 27 weeks. Histological scoring in WT and *METTL3*^{LKI} mice fed normal diet at 27 weeks. Data are presented as mean ± SD. Statistical significance was determined by two-sided Student's *t*-test.

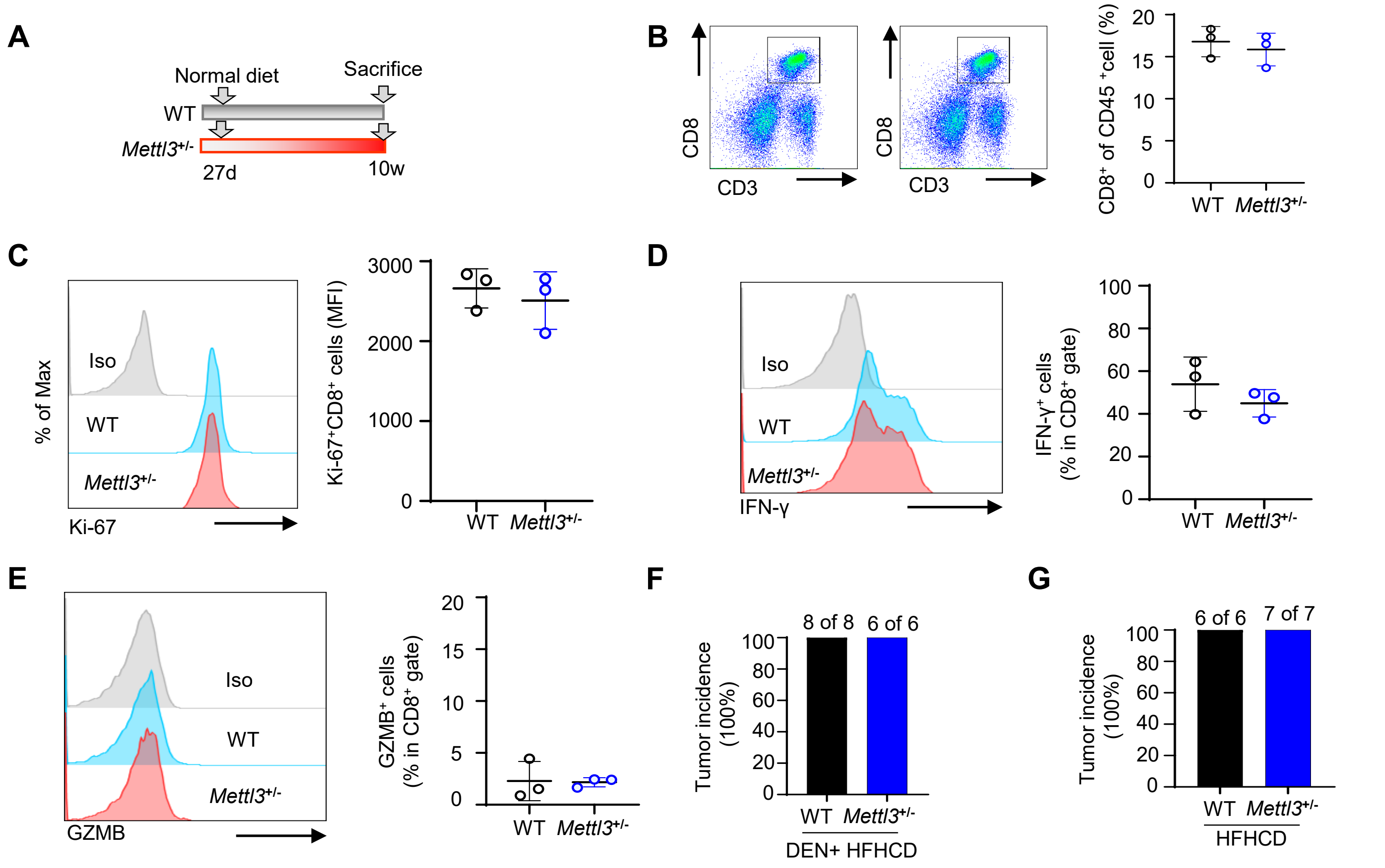

Figure S3. METTL3 knockout suppresses diet-induced NAFLD-HCC. Related to Figure 3. (**A**) Experimental schematic of WT and *Mettl3*$^{+/-}$ mice were fed with normal diet (ND) for 10 weeks (n=3/group). (**B**) Infiltration of CD8^{+} T cells in spleen from WT and *Mettl3*$^{+/-}$ mice treated with ND by flow cytometry. (**C**) Infiltration of ki-67^{+} CD8^{+} T cells in spleen from WT and *Mettl3*$^{+/-}$ mice treated with ND by flow cytometry. (**D and E**) Infiltration of IFN-γ^{+} (D) and GZMB^{+} (E) CD8^{+} T cells in spleen from WT and *Mettl3*$^{+/-}$ mice fed ND by flow cytometry.(**F**) NAFLD-HCC tumor incidence in WT and *Mettl3*$^{+/-}$ mice treated with DEN plus HFHCD. (**G**) NAFLD-HCC tumor incidence in WT and *Mettl3*$^{+/-}$ mice treated with HFHCD. Data are presented as mean ± SD. Statistical significance was determined by two-sided Student's *t*-test.

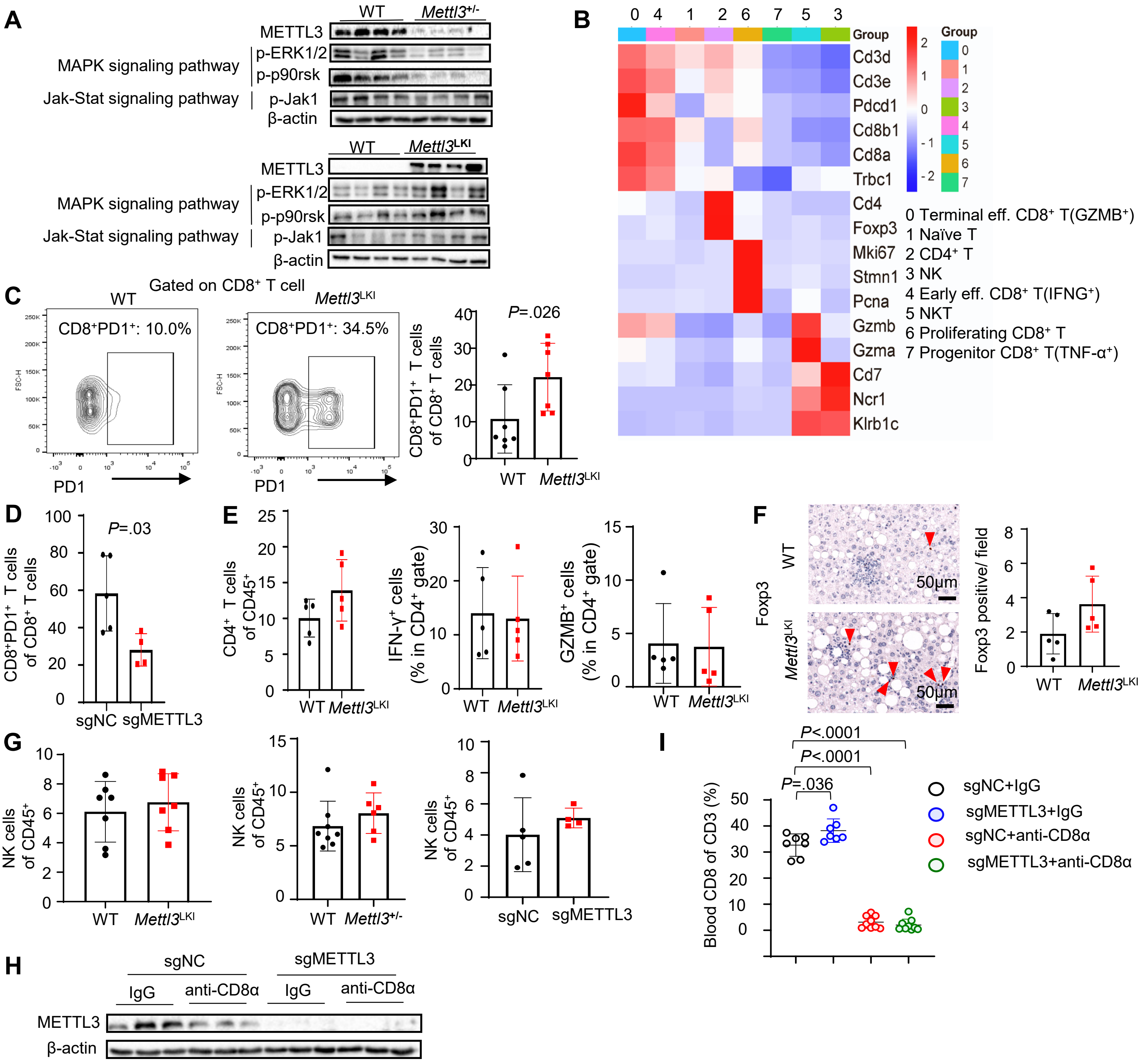

Figure S4. Tumor-intrinsic METTL3 restricts the activation and effector states of CD8⁺ T cells. Related to Figure 4. (**A**) Detection of MAPK signaling pathway and Jak-Stat signaling pathway by Western blot in WT and *Mettl3*$^{+/-}$ mice fed HFHCD (*upper*), also WT and *Mettl3*^{LKI} mice treated with DEN plus CDHFD (*bottom*). (**B**) Gene markers for different clusters of tumor-infiltrating T/NK-cells population. (**C**) Proportion of $CD8^+$ $PD1^+$ T cells (among $CD8^+$ T cells) in WT and *Mettl3*^{LKI} mice treated with DEN plus HFHCD by flow cytometry. (**D**) Proportion of $CD8^+$ $PD1^+$ T cells (among $CD8^+$ T cells) in NAFLD-HCC orthotopic mice model with sgNC and sgMETTL3 in Hepa1-6 by flow cytometry. (**E**) Proportion of $CD4^+$ T cells (among $CD45^+$ T cells) and its function markers (among $CD4^+$ T cells) in tumors from WT and *Mettl3*^{LKI} mice treated with DEN plus CDHFD by flow cytometry. (**F**) Foxp3 staining of livers of WT and *Mettl3*^{LKI} mice. (**G**)Tumor infiltration of NK cells in different models by flow cytometry, WT and *Mettl3*^{LKI} mice treated with DEN plus CDHFD (*left*), WT and *Mettl3*$^{+/-}$ mice treated with DEN plus HFHCD (*middle*), NAFLD-HCC orthotopic mice model with sgNC and sgMETTL3 in Hepa1-6 by flow cytometry (*right*). (**H**) Knockdown efficiency of METTL3 in orthotopic tumors by western blot. (**I**) Proportion of $CD8^+$ T cells (among $CD3^+$ T cells) in blood by flow cytometry. Successfully depletion of $CD8^+$ T cells using anti-CD8α compared to IgG group. Data are presented as mean ± SD. Statistical significance was determined by one-way ANOVA or two-sided Student's *t*-test where appropriate.

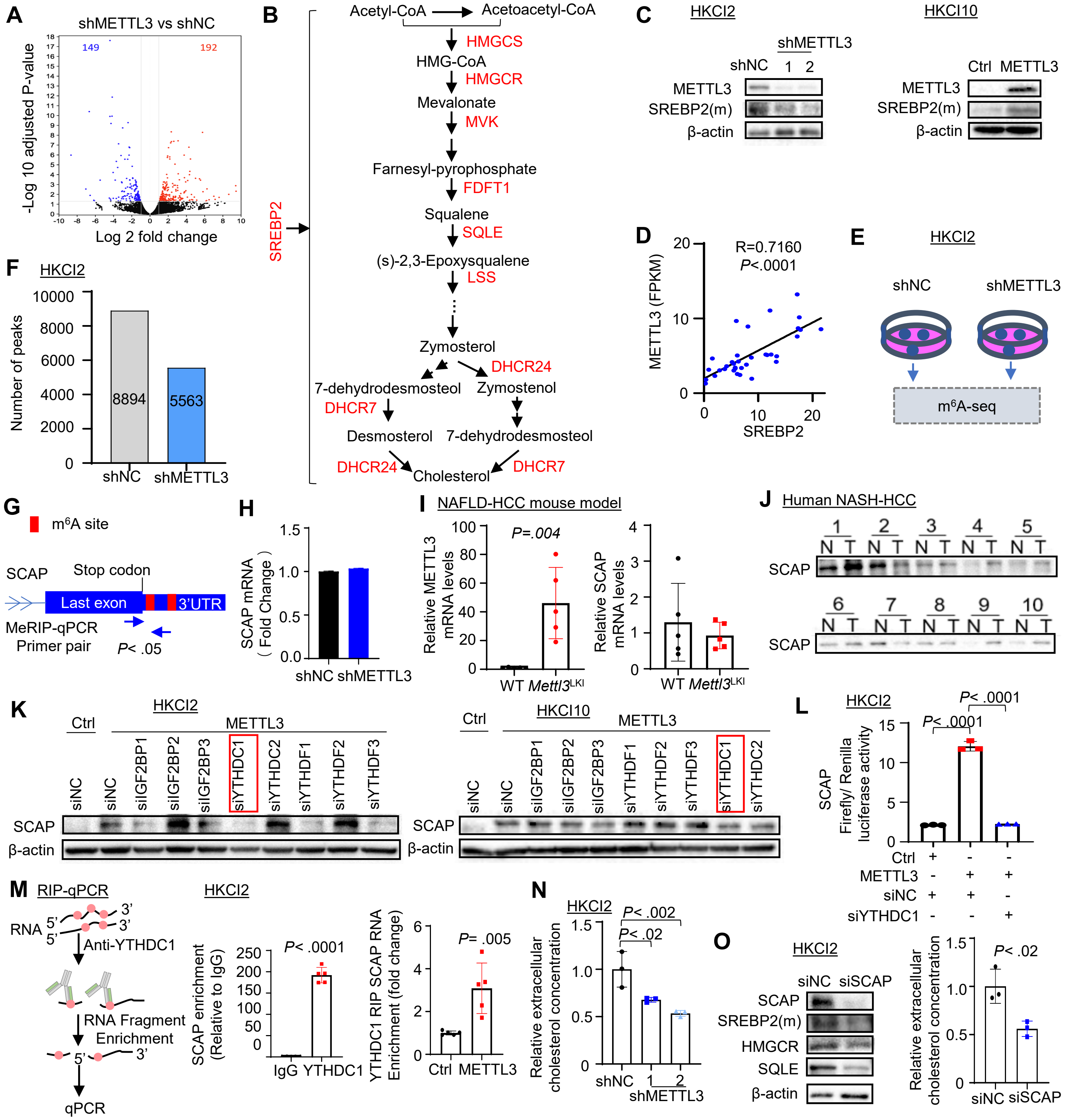

Figure S5. SCAP serves as a target of METTL3 via an YTHDC1-dependent mechanism in NAFLD-HCC. Related to Figure 5 and Table S1. (**A**) RNA-seq showed that 149 genes were down-regulated when METTL3 was silenced in HKCI2 cells. (**B**) Cholesterol biosynthesis pathway related genes were down regulated when METTL3 knockdown in HKCI2 cells by RNA-seq (in red). (**C**) Mature SREBP2 protein expression in HKCI2 cells with METTL3 knockdown, and HKCI10 cells with METTL3 overexpression. (**D**) Correlation of METTL3 with SREBP2 in human NAFLD-HCC. (**E**) Flow chart for m^{6}A-seq of NAFLD-HCC cells (HKCI2) with or without METTL3 knockdown. (**F**) m^{6}A-seq of HKCI2 showed a number of m^{6}A peaks. (**G**) Scheme showing the design of primers for MeRIP-qPCR to validate m^{6}A modifications on SCAP mRNA. Potential m^{6}A sites were highlighted in red, from 47413830 to 47413930 (*P*< .05 in m^{6}A-seq), and 94419499 to 47419600 (*P*>.05). Primers used in MeRIP-qPCR to validated m^{6}A modification are indicated by arrows in blue. (**H**) No change in SCAP mRNA by METTL3 knockdown in HKCI2 cells by RNA-seq. (**I**) METTL3 mRNA level was upregulated in *METTL3*^{LKI} mice compared to WT mice by qPCR, while SCAP mRNA expression was unchanged. (**J**) SCAP protein expression in human NAFLD-HCC was validated in an independent cohort (n=10). Loading control is identical to Figure 1C. (**K**) Screening potential m^{6}A reader for SCAP mediated by METTL3 in HKCI2 and HKCI10 by western blot. (**L**) Luciferase activity of SCAP 3-UTR in HKCI2 cells upon METTL3 overexpression with or without siYTHDC1, relative to Renilla luciferase activity. (**M**) YTHDC1-RIP-qPCR in HKCI2 cells. Schematic of YTHDC1-RIP-qPCR in HKCI2 cells (*left*). Relative enrichment of SCAP RNA by YTHDC1 in HKCI2 cells (*middle*). Relative enrichment of SCAP upon METTL3 overexpression in HKCI2 by YTHDC1-RIP-qPCR (*right*). (**N**) Relative extracellular cholesterol concentration upon METTL3 knockdown. (**O**) Cholesterol biosynthesis genes protein was suppressed in HKCI2-siSCAP cells (*left*), and relative extracellular cholesterol concentration upon SCAP knockdown (*right*) in HKCI2. Data are presented as mean ± SD. Statistical significance was determined by one-way ANOVA or two-sided Student's *t*-test where appropriate.

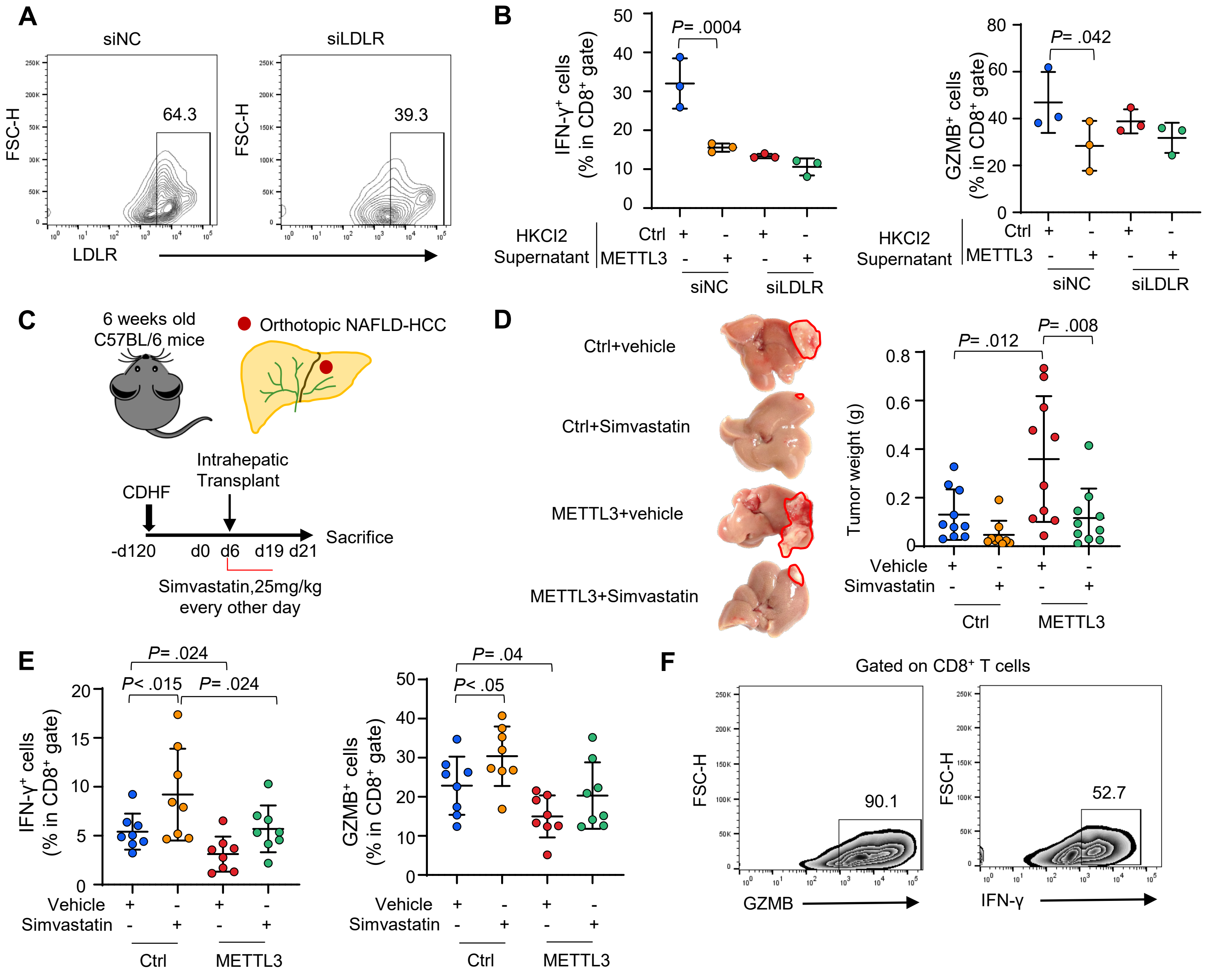

Figure S6. METTL3-induced cholesterol functions as an inducer of immunosuppression in NAFLD-HCC microenvironment. Related to Figure 6 and Table S1. (**A**) Knockdown efficiency of siLDLR in human T cells by flow cytometry. (**B**) Quantification of human IFN-γ$^+$ and GZMB$^+$ CD8$^+$ T cells from different groups by flow cytometry. (**C**) Experimental design for the orthotopic NAFLD-HCC model and simvastatin treatment. (**D**) Representative images of mouse livers and tumor weight at end point (n=10/group) . (**E**) Quantification of tumor-infiltrating IFN-γ$^+$ and GZMB$^+$ CD8$^+$ T cells. (**F**) Expression of IFN-γ$^+$ and GZMB$^+$ CD8$^+$ T cells by flow cytometry before coculture. Data are presented as mean ± SD. Statistical significance was determined by one-way ANOVA or two-sided Student's *t*-test where appropriate.

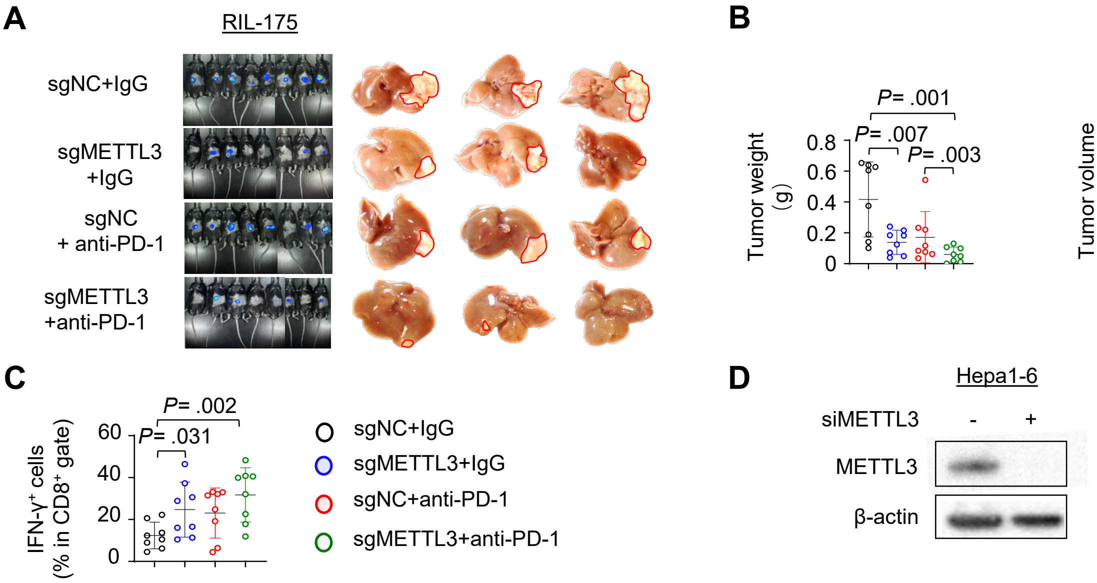

Figure S7. METTL3 is a therapeutic target for NAFLD-HCC. Related to Figure 7. (A) C57BL/6J mice were inoculated with 5x10^5 RIL175-sgNC and RIL175-sgMETTL3 cells, and then treated with anti-PD1 or IgG control. Bioluminescent imaging and the representative gross morphology of livers. **(B)** Tumor weight (left) and volume (right) at end point (n=8/group). **(C)** Quantification of tumor-infiltrating IFN-γ^+ $CD8^+$ T cell from different groups. **(D)** Knockdown efficiency of METTL3 in Hepa1-6 by nanoparticle siRNA was determined by western blot. Data are presented as mean ± SD. Statistical significance was determined by one-way ANOVA or two-sided Student's *t*-test where appropriate.

Table S1. Oligonucleotides used in this study. Related to STAR Methods.

REAGENT or RESOURCE	SOURCE	IDENTIFIER
Oligonucleotides		
Human SCAP for Merip-qPCR<br>Forward: TGGTGTATGTGCCCTCTGTG<br>Reverse: CCCCAAGTCCAGGTTCAGT	This paper	In this study
Human β-actin for RT-PCR<br>Forward: CACCATTGGCAATGAGCGGTTC<br>Reverse: AGGTCTTTGCGGATGTCCACGT	This paper	In this study
Mouse β-actin for RT-PCR<br>Forward: CATTGCTGACAGGATGCAGAAGG<br>Reverse: TGCTGGAAGGTGGACAGTGAGG	This paper	In this study
Human METTL3 for RT-PCR<br>Forward: CTATCTCCTGGCACTCGCAAGA<br>Reverse: GCTTGAACCGTGCAACCACATC	This paper	In this study
Mouse Mettl3 for RT-PCR<br>Forward: CAGTGCTACAGGATGACGGCTT<br>Reverse: CCGTCCTAATGATGCGCTGCAG	This paper	In this study
SiRNA for YTHDF1-1<br>Forward: CCUCCACCCAUAAAGCAUATT<br>Reverse: UAUGCUUUAUGGGUGGAGGTT	This paper	In this study
SiRNA for YTHDF1-2<br>Forward: GCUCCAUUAAGUACUCCAUTT<br>Reverse: AUGGAGUACUUAAUGGAGCTT	This paper	In this study
SiRNA for YTHDF2-1<br>Forward: GCUCUGGAUAUAGUAGCAATT<br>Reverse: UUGCUACUAUAUCCAGAGCTT	This paper	In this study
SiRNA for YTHDF2-2<br>Forward: GCGGGUCCAUUACUAGUAATT<br>Reverse: UUACUAGUAAUGGACCCGCTT	This paper	In this study
SiRNA for YTHDF3-1<br>Forward: GGGACAAUCAACACAAAGUTT<br>Reverse: ACUUUGUGUUGAUUGUCCCTT	This paper	In this study
SiRNA for YTHDF3-2<br>Forward: GACUAGCAUUGCAACCAAUTT<br>Reverse: AUUGGUUGCAAUGCUAGUCTT	This paper	In this study
SiRNA for YTHDC1-1<br>Forward: GCUCUGCAUCAGAGUCAUATT<br>Reverse: UAUGACUCUGAUGCAGAGCTT	This paper	In this study
SiRNA for YTHDC1-2<br>Forward: GCAAGGAGUGUUAUCUUAATT<br>Reverse: UUAAGAUAACACUCCUUGCTT	This paper	In this study
SiRNA for YTHDC2-1<br>Forward: GCAGAGAACUGUUCUAAAUTT<br>Reverse: AUUUAGAACAGUUCUCUGCTT	This paper	In this study
SiRNA for YTHDC2-2<br>Forward: CCUGUUCGAUACUUCAUAATT<br>Reverse: UUAUGAAGUAUCGAACAGGTT	This paper	In this study
SiRNA for IGF2BP1-1<br>Forward: CCCAGUAUGUGGGUGCCAUTT<br>Reverse: AUGGCACCCACAUACUGGGTT	This paper	In this study
SiRNA for IGF2BP1-2<br>Forward: CCAAAGUUCGUAUGGUUAUTT<br>Reverse: AUAACCAUACGAACUUUGGTT	This paper	In this study
SiRNA for IGF2BP2-1<br>Forward: GUCAACGUCACAUAUGCAATT<br>Reverse: UUGCAUAUGUGACGUUGACTT	This paper	In this study

SiRNA for IGF2BP2-2<br>Forward: GAGGGCUUGACCAUAAAGATT<br>Reverse: UCUUUAUGGUCAAGCCCUCTT	This paper	In this study
SiRNA for IGF2BP3-1<br>Forward: GGAUUCGGAAACUUCAGAUTT<br>Reverse: AUCUGAAGUUUCCGAAUCCTT	This paper	In this study
SiRNA for IGF2BP3-2<br>Forward: CACCUUGAAAGUAGCCUAUTT<br>Reverse: AUAGGCUACUUUCAAGGUGTT	This paper	In this study
Mouse Mettl3 siRNA<br>Forward: CCAAGGAAGAGUGCAUGAATT<br>Reverse: ACUCUUCCUUGGTT	This paper	In this study
SiNC:<br>Forward: UUCUCCGAACGUGUCACGUTT<br>Reverse: ACGUGACACGUUCGGAGAATT	This paper	In this study
Negative control siRNA	Invitrogen	Cat#4390843
Human LDLR siRNA-1<br>Forward: GCGACAGAUGCGAAAGAAATT<br>Reverse: UUUCUUUCGCAUCUGUCGCTT	This paper	In this study
Human LDLR siRNA-2<br>Forward: ACGGGAAAUGCAUCUCCUATT<br>Reverse: UAGGAGAUGCAUUUCCCGUTT	This paper	In this study
Human LDLR siRNA-3<br>Forward: GCUCUGAUGAGUCCCAGGATT<br>Reverse: UCCUGGGACUCAUCAGAGCTT	This paper	In this study
